# Host preference and invasiveness of commensal bacteria in the *Lotus* and *Arabidopsis* root microbiota

**DOI:** 10.1038/s41564-021-00941-9

**Published:** 2021-07-26

**Authors:** Kathrin Wippel, Ke Tao, Yulong Niu, Rafal Zgadzaj, Niklas Kiel, Rui Guan, Eik Dahms, Pengfan Zhang, Dorthe B. Jensen, Elke Logemann, Simona Radutoiu, Paul Schulze-Lefert, Ruben Garrido-Oter

**Affiliations:** 1 Department of Plant-Microbe Interactions, Max Planck Institute for Plant Breeding Research, Cologne, Germany; 2 Department of Molecular Biology and Genetics, Faculty of Science and Technology, Aarhus University, Aarhus, Denmark; 3 Cluster of Excellence on Plant Sciences, Düsseldorf, Germany

**Keywords:** Microbiome, Plant sciences

## Abstract

Roots of different plant species are colonized by bacterial communities, that are distinct even when hosts share the same habitat. It remains unclear to what extent the host actively selects these communities and whether commensals are adapted to a specific plant species. To address this question, we assembled a sequence-indexed bacterial culture collection from roots and nodules of *Lotus japonicus* that contains representatives of most species previously identified using metagenomics. We analysed taxonomically paired synthetic communities from *L. japonicus* and *Arabidopsis thaliana* in a multi-species gnotobiotic system and detected signatures of host preference among commensal bacteria in a community context, but not in mono-associations. Sequential inoculation experiments revealed priority effects during root microbiota assembly, where established communities are resilient to invasion by latecomers, and that host preference of commensal bacteria confers a competitive advantage in their cognate host. Our findings show that host preference in commensal bacteria from diverse taxonomic groups is associated with their invasiveness into standing root-associated communities.

## Abstract (web summary)

Host preferences of commensal bacteria in the root microbiota are revealed using systematic analyses of synthetic bacterial communities in a gnotobiotic system.

## Main

Plant roots associate with diverse microorganisms that are recruited from the surrounding soil biome and that assemble into structured communities known as the root microbiota. These communities provide the host with beneficial functions, such as indirect pathogen protection or mineral nutrient mobilization^1–3^. Despite conservation at higher taxonomic ranks^4–7^, comparison of community profiles across diverse land plants shows a clear separation according to host species^5,7^. These patterns could be explained by a process in which the root microbiota assemble according to niches defined by plant traits that in turn diversify as a result of plant adaptation to their environment. Alternatively, variation of microbiota profiles along the host phylogeny may be at least partially caused by coadaptation between the plant and its associated microbial communities.

Culture-independent amplicon sequencing allows characterization of community structures and taxonomic composition but does not allow the study of phenotypes of individual community members. To overcome this fundamental limitation in microbiota studies, comprehensive culture collections of sequenced strains isolated from root and leaf tissue have been established^2,3,8,9^. Synthetic communities (SynComs) built from these collections can be used in gnotobiotic reconstitution systems of reduced complexity to explore the role of immune signalling^10^, nutritional status^3,11^, biotic and abiotic stress^2^ and priority effects^12^ in the establishment of the root and leaf microbiota.

To investigate plant host preference of commensal bacteria, we assembled a collection of cultured bacterial species from the roots and nodules of the model legume *Lotus japonicus* (hereafter *Lj*) that is comparable to the collection previously established from *Arabidopsis thaliana* (hereafter *At*) roots^8^ in terms of taxonomic and genomic composition, despite 125 Myr of divergence between *Lj* and *At*^13^ whose crown groups evolved 65 and 32 Mya, respectively^14^. These two collections originate from plants grown in the same soil, enabling us to design SynComs for microbiota reconstitution experiments. Using this setup, we investigated host preference of commensal communities and the role of nitrogen-fixing nodule symbiosis, immunity and root exudation in microbiota establishment.

## Results

### Host-species-specific bacterial culture collections

**Fig. 1:**
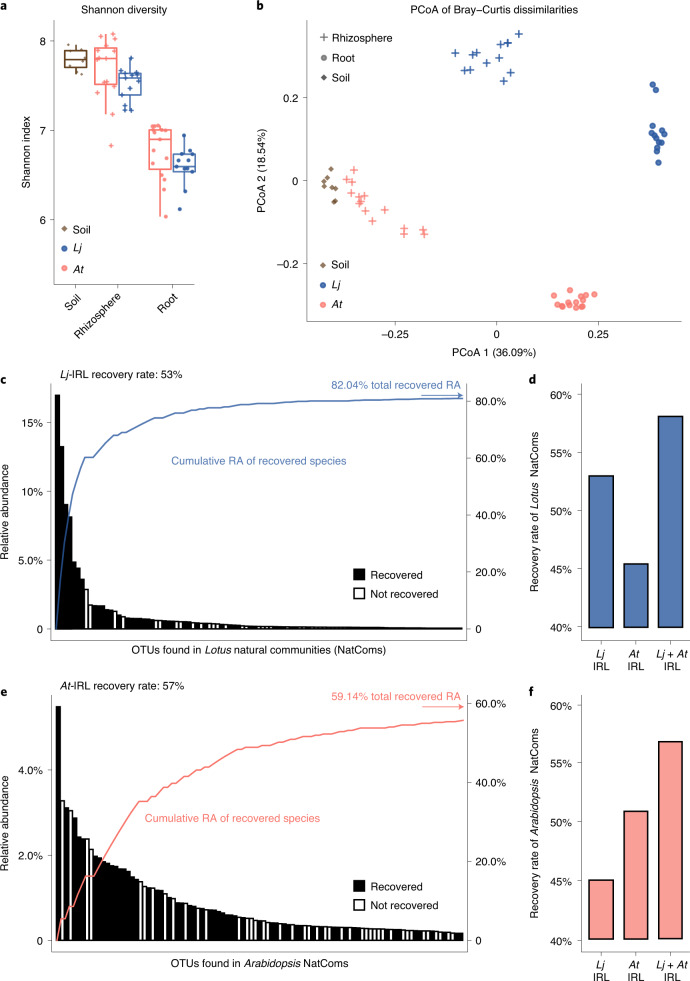
*Lotus* and *Arabidopsis* root-associated bacterial communities. **a**, Alpha diversity analysis of soil- (*n* = 8), rhizosphere- (*n* = 13 for Gifu, *n* = 15 for Col-0) and root-associated bacterial communities (*n* = 13 for Gifu, *n* = 15 for Col-0) from *Lj* and *At* plants grown in natural soil (exp. A), assessed using the Shannon index. **b**, PCoA of Bray–Curtis dissimilarities of the same communities (*n* = 64). **c**,**e**, Rank abundance plots of OTUs found in the *Lotus* (**c**) and *Arabidopsis* (**e**) natural root communities. Community members captured in the corresponding culture collection are depicted as black while non-recovered OTUs are shown in white. The vertical axis on the right shows the accumulated relative abundance in natural communities of all recovered OTUs. **d**,**f**, Percentage of abundant OTUs (≥0.1% RA) associated with *Lotus* (**d**) or *Arabidopsis* (**f**) roots in nature (natural communities, NatComs) that are captured in the *Lotus* or the *Arabidopsis* IRLs (*At-* and *Lj*-IRL). Source data

**Extended Data Fig. 1:**
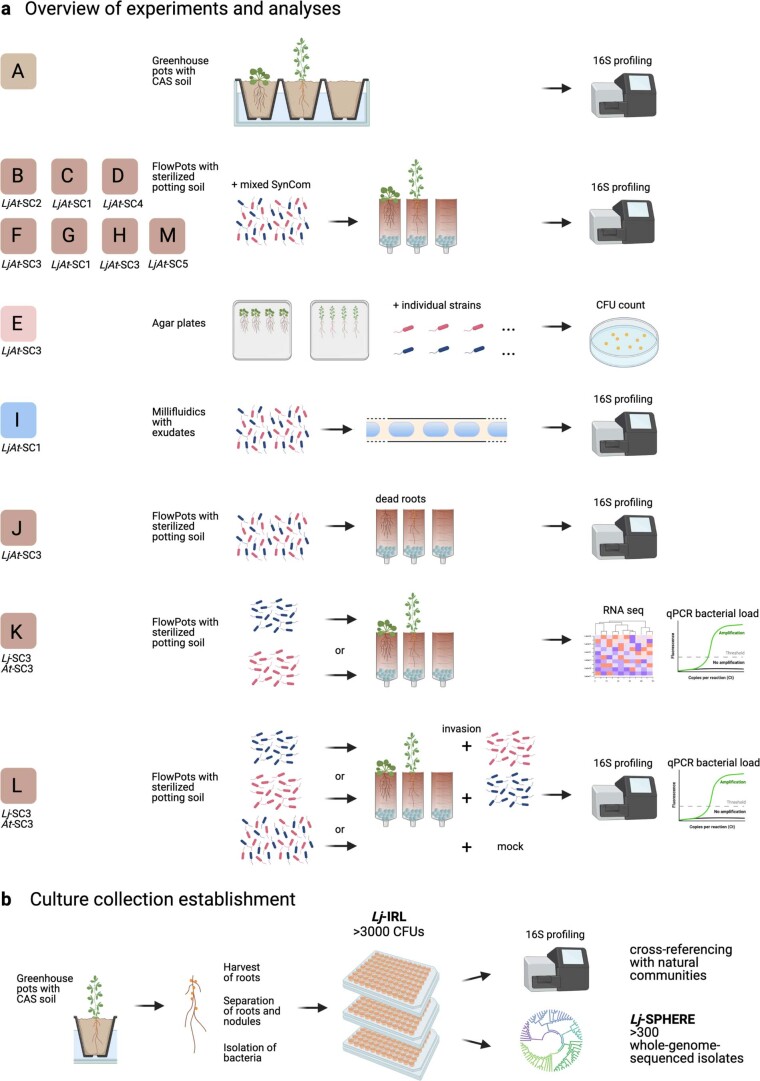
Flow chart of experimental procedures. **a**, Overview of experiments and type of analyses performed on root, rhizosphere, and soil samples, or millifluidic droplets. **b**, Culture collection establishment of *Lotus japonicus* root and nodule bacterial isolates. Image created with BioRender.com.

**Extended Data Fig. 2:**
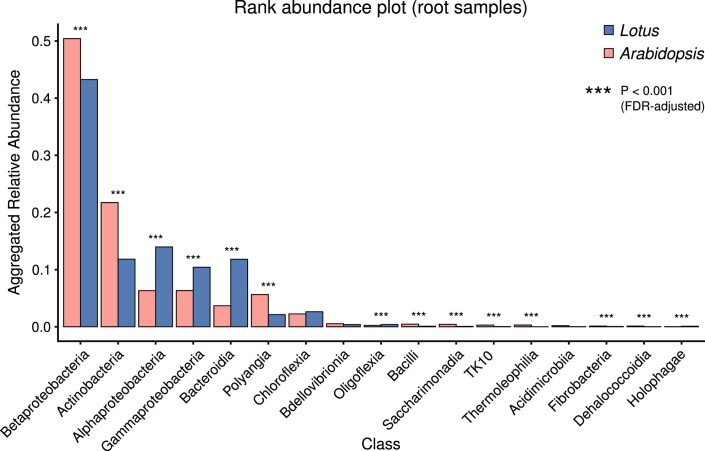
Culture-independent diversity analysis of root-associated bacterial communities from *Lotus* and *Arabidopsis*. Rank abundance plot of bacterial communities from *Lotus* or *Arabidopsis* roots, aggregated to the class level, including all families with a mean accumulated relative abundance of > 0.1% on either host. Statistical differences were assessed using a two-sided, non-parametric Mann–Whitney test. Asterisks represent significant values after multiple testing correction using the Benjamini–Hochberg method (*P* < 0.05).

We compared the bacterial communities associated with roots of *Lj* and *At* plants grown in the same soil (experiment (exp.) A, Extended Data Fig. 1a and Supplementary Table 2)^2,3,15^ and confirmed that both hosts associate with communities that are clearly distinct from those of the surrounding soil (Fig. 1a,b). This shift is characterized by a decrease in alpha diversity (within-sample diversity; Fig. 1a) as well as by a separation between root, rhizosphere and soil samples (beta diversity; Fig. 1b, principal coordinates analysis (PCoA) 1). In addition, *Lj* and *At* root samples formed two distinct clusters, indicating host-species-specific recruitment of commensals from identical pools of soil-dwelling bacteria (Fig. 1b, PCoA 2), which is in line with previous studies^15,16^. This separation (28% of variance, *P* = 0.001) was mainly explained by the different relative abundance of Proteobacteria, Actinobacteria, Bacteroidetes (Flavobacteria and Sphingobacteria) and Firmicutes (Bacilli) in *Lj* compared to *At* (Extended Data Fig. 2).

To explore the mechanisms by which different plant species associate with distinct microbial communities, we established a taxonomically and functionally diverse culture collection of the *Lj* root and nodule microbiota (Extended Data Fig. 1b). A total of 3,960 colony-forming units were obtained and taxonomically characterized by sequencing the bacterial 16S rRNA (Supplementary Data 1), resulting in a comprehensive sequence-indexed rhizobacterial library (IRL) from *Lj* (*Lj*-IRL). In parallel, a subset of the root samples was also subjected to amplicon sequencing to obtain culture-independent community profiles for cross-referencing with the *Lj*-IRL data. In the *Lj* collection, we were able to recover up to 53% of the most abundant bacterial operational taxonomic units (OTUs, defined by 97% 16S rRNA sequence identity) found in the corresponding natural community profiles, compared with 57% for the *At* collection (Fig. 1c,e and Supplementary Note). The recovered bacterial taxa in the respective collection accounted for 82% of all sequencing reads from *Lj* root samples and 59% from *At*. Approximately 45% of the abundant OTUs found in the natural communities of one host were captured in the culture collection of the other species (Fig. 1d,f), indicating a substantial overlap of the recovered bacterial taxa. Both culture collections include members of the Actinobacteria, Proteobacteria, Bacteroidetes and Firmicutes, the four phyla robustly found in the root microbiota of diverse plant species^5,7^.

**Extended Data Fig. 3:**
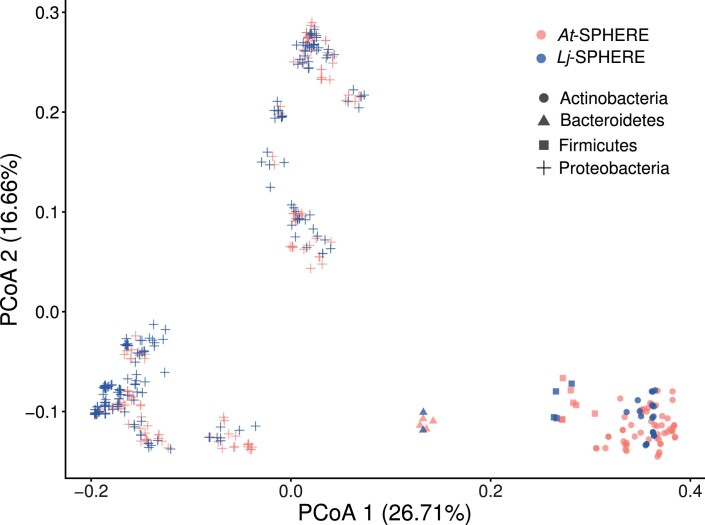
Functional overlap of *Lotus* and *Arabidopsis* culture collection genomes. PCoA of functional distances of genomes from bacterial isolates of the *Lotus* (*Lj*-SPHERE; *n* = 294) and the *Arabidopsis* (*At*-SPHERE; *n* = 194) culture collections.

To establish a core *Lj* culture collection of whole-genome sequenced strains (*Lj*-SPHERE), we selected from the *Lj*-IRL a taxonomically representative subset of bacterial isolates maximizing the number of covered taxa, as previously done for *At* (Methods)^8^. A total of 294 isolates belonging to 20 families and 124 species, including both commensal and mutualistic bacteria, were subjected to whole-genome sequencing (WGS) (Supplementary Data 2). Comparative analyses of all sequenced isolates from both collections revealed an extensive taxonomic and genomic overlap between exemplars derived from *Lj* and *At* (Extended Data Fig. 3 and Supplementary Note). This indicates that the observed differences in natural community structures (Fig. 1b) are probably not driven by the presence of host-specific bacterial taxonomic groups. Instead, the distinct root community profiles of the two hosts are possibly due to differences in the relative abundance of shared taxonomic groups (Extended Data Fig. 2).

### Host preference of commensal synthetic communities

**Fig. 2:**
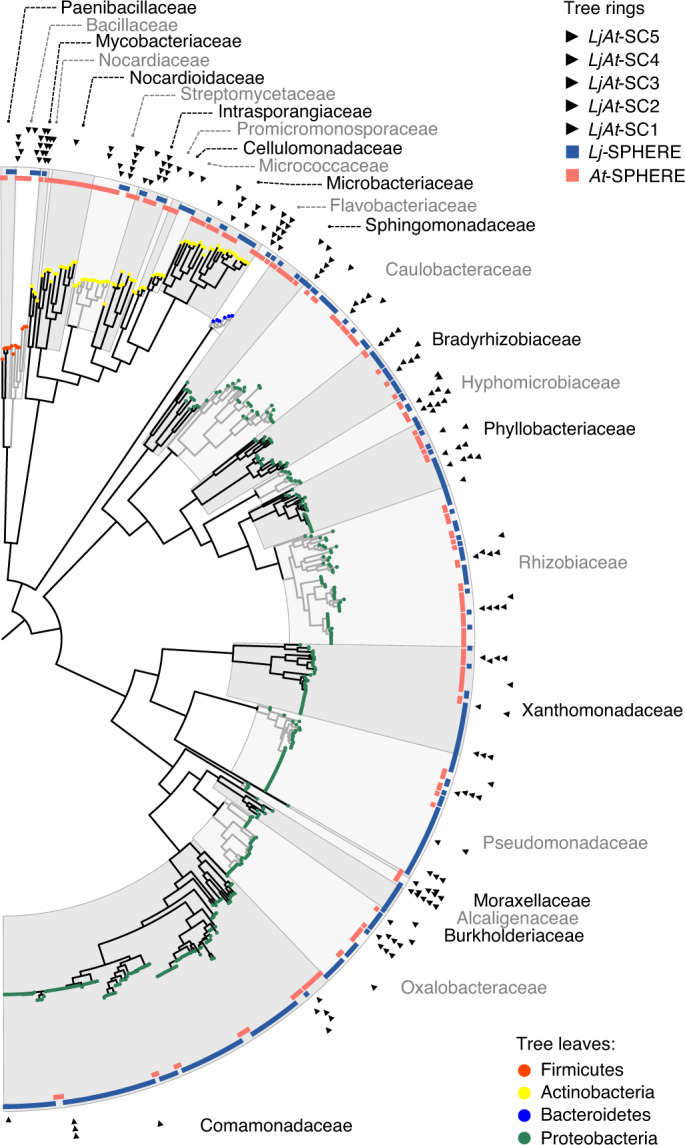
Whole-genome phylogeny of the *Lotus* and *Arabidopsis* core culture collections. Maximum likelihood phylogeny, constructed from a concatenated alignment of 31 conserved, single-copy genes (AMPHORA) showing the taxonomic overlap of the *Lj*-SPHERE (*n* = 294, blue track) and *At*-SPHERE (*n* = 194, red track) core culture collections. Arrows in the outer rings indicate the strains selected for five mixed communities used in reconstitution experiments. Source data

**Fig. 3:**
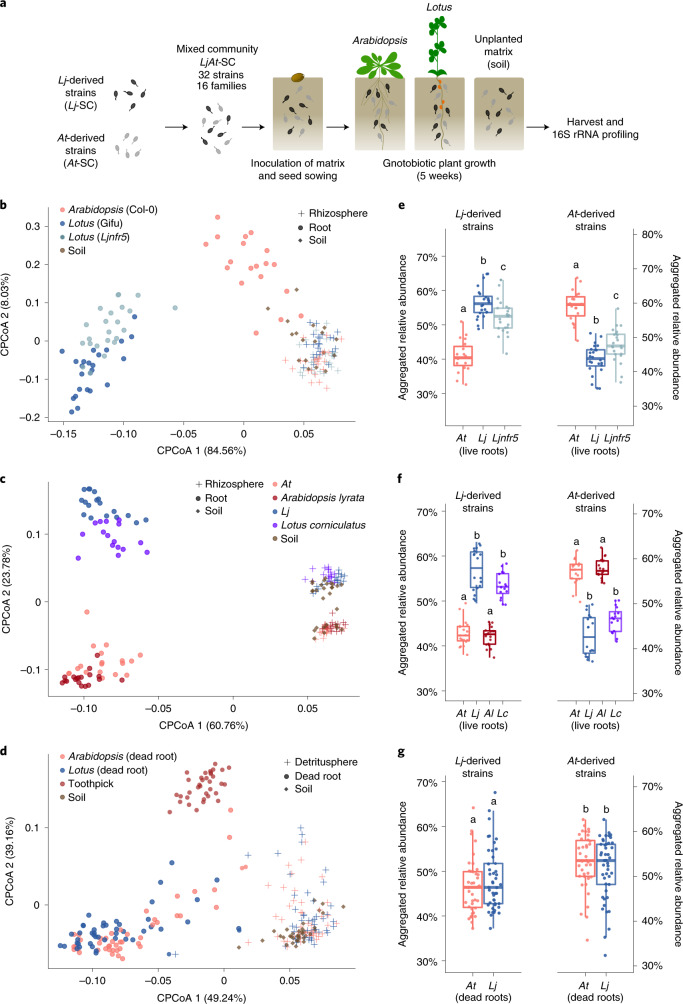
Reconstitution experiments recapitulate culture-independent patterns and show signatures of host preference by commensal communities. **a**, Setup of the competition experiments. **b**–**d**, Constrained PCoA (CPCoA) of Bray–Curtis dissimilarities (constrained by all biological factors and conditioned by all technical variables) of soil, rhizosphere and root samples. **b**, *Lj* wild-type Gifu, *nfr5* mutant and *At* wild-type Col-0 plants cocultivated with the mixed community *LjAt*-SC2 (exp. B, *n* = 155, variance explained 53.8%, *P* = 0.001). **c**, Gifu, Col-0, *A. lyrata* MN47 (*Al*) and *L. corniculatus* cocultivated with *LjAt*-SC3 (exp. F, *n* = 173, variance explained 65.1%, *P* = 0.001). **d**, Dead roots of Gifu and Col-0, and toothpick cocultivated with *LjAt*-SC3 (exp. J, *n* = 250, variance explained 43.9%, *P* = 0.001). **e**–**g**, Aggregated RA of the 16 *Lj*-derived and the 16 *At*-derived strains in the live (**e**,**f**) or dead roots (**g**) of *Lotus* and *Arabidopsis* plants inoculated with *LjAt*-SC2 (*n* = 66, **e**) or *LjAt*-SC3 (*n* = 72, **f** and *n* = 89, **g**). Source data

**Extended Data Fig. 4:**
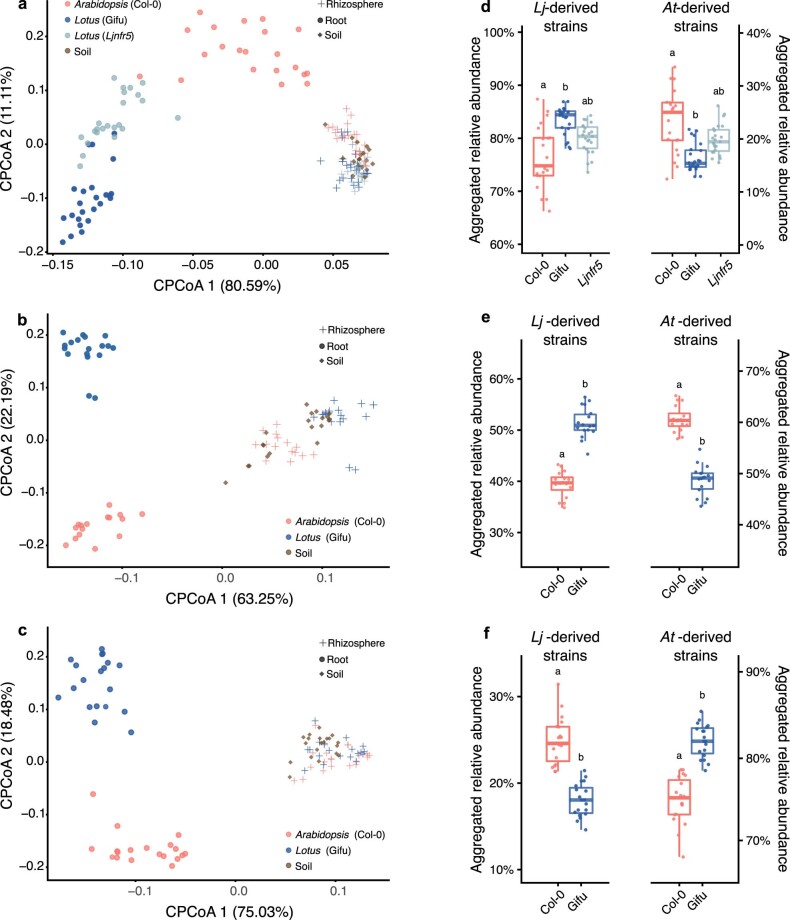
Host-species specific bacterial root communities and commensal host preference is confirmed using independent mixed communities. **a** and **c**, Constrained PCoA of Bray-Curtis dissimilarity (constrained by all biological factors and conditioned by all technical variables) of soil, rhizosphere, and root samples from *L. japonicus* wild-type Gifu, *nfr5* mutant, and *A. thaliana* wild type Col-0 plants co-cultivated with the mixed community *LjAt*-SC1 (**a**, experiment C, *n* = 155, variance explained 53.8%, *P* = 0.001), from Gifu and Col-0 co-cultivated with *LjAt*-SC4 (**b**, experiment D, *n* = 87, variance explained 60%, *P* = 0.001), or from Gifu and Col-0 co-cultivated with *LjAt*-SC5 (**b**, experiment M, *n* = 100, variance explained 67%, *P* = 0.001). **d**, **e** and **f**, Aggregated RA of the 16 *Lj*-derived and the 16 *At*-derived strains in the roots of *Lotus* and *Arabidopsis* plants inoculated with *LjAt*-SC1 (**d;** *n* = 68), *LjAt*-SC4 (**e**; *n* = 34), or *LjAt*-SC5 (**f**; *n* = 40). *n* refers to biologically independent samples.

Given the overlap between the *Lj*- and *At*-SPHERE culture collections at a high taxonomic and whole-genome level, we speculated that strain-specific phenotypic variation in planta could allow commensal bacteria to preferentially colonize their cognate host. To test this hypothesis, we designed taxonomically paired SynComs for each host, representing 16 bacterial families present in both collections (Fig. 2). We then combined these SynComs into a mixed community composed of 32 strains (Supplementary Table 1). We allowed commensal bacteria to compete for colonization of the host from which they were derived (hereafter referred to as native strains) with strains isolated from the other plant species (non-native strains, Supplementary Fig. 1a). We used a gnotobiotic system^2,17^ to grow wild-type *At* (Col-0), *Lj* (Gifu) and a *Lj* mutant deficient in root nodule symbiosis (*Ljnfr5*)^18^ in the presence of the mixed community (Fig. 3a). After 5 weeks, we performed community profiling via 16S rRNA gene amplicon sequencing of the root, rhizosphere and unplanted soil compartments. Analysis of community diversity revealed a significant separation (*P* = 0.001) of communities of root samples from those of rhizosphere and soil, which in turn clustered together (exp. B, Fig. 3b). In addition, we observed that the two hosts are colonized by distinct root microbial communities starting from the same input, and that samples from wild-type *Lj* are differentiable from those of *Ljnfr5* (Fig. 3b). These results were confirmed by two independent, full factorial experiments using different mixed communities (exp. C and M, Extended Data Fig. 4a,c). An additional experiment, where strains belonging to families found exclusively in the *Lj* or *At* culture collections (two and five families, respectively) were added to the mixed community, resulted in similar patterns of beta diversity (exp. D, Extended Data Fig. 4b). These results recapitulate the community shifts between compartment, host species and plant genotype, which were previously observed in culture-independent community profiles obtained from plants grown in natural soils (Fig. 1b)^4,6,15,16^, thus validating our comparative reconstitution system to study host-species-specific microbiota establishment.

**Extended Data Fig. 5:**
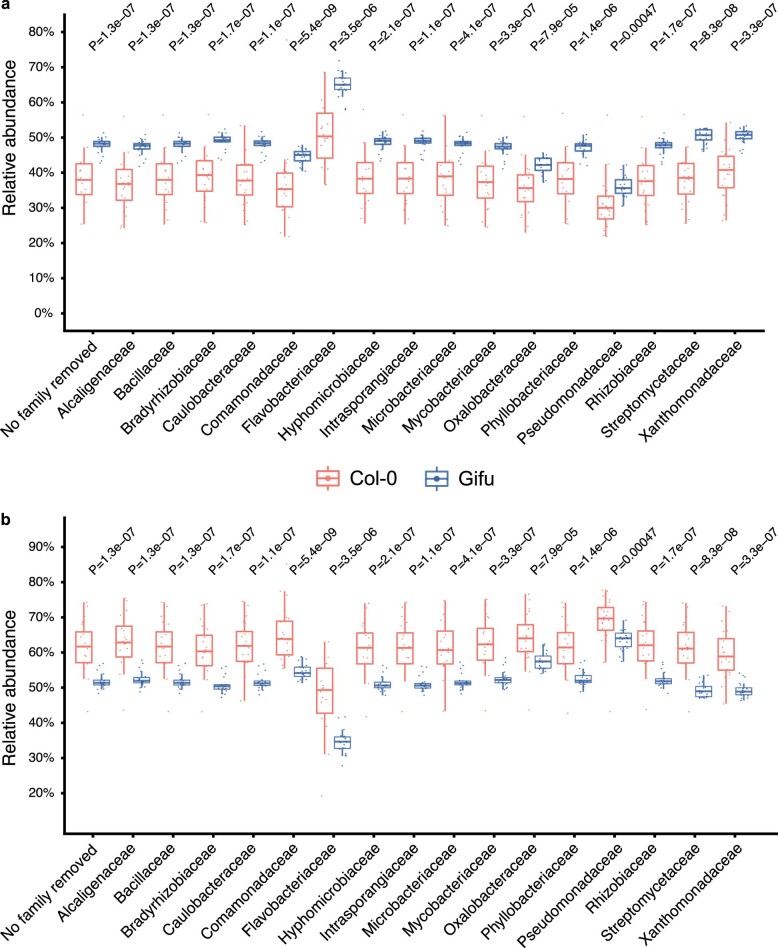
Host preference is retained after *in silico* removal of individual bacterial families. Aggregated relative abundance of *Lotus*- (**a**, *n* = 20) and *Arabidopsis*-derived (**b**, *n* = 16) strains in roots from plants inoculated with the mixed community *LjAt*-SC3 (experiment L). Host preference was assessed using a Mann-Whitney non-parametric test after *in silico* removal of each family. The x-axis labels indicate each depleted family. *n* refers to biologically independent samples.

**Extended Data Fig. 6:**
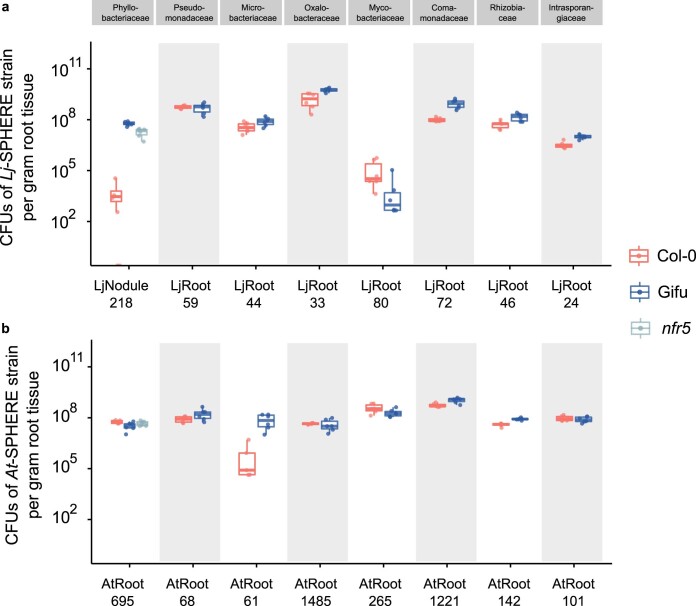
Bacterial abundance in mono-association with host plants. Bacterial abundances of commensal bacteria (strain IDs indicated at *x*-axis) colonizing Col-0, Gifu, and *nfr5* roots, assessed by counting of colony forming units (CFUs) after extraction from root tissue. Plants were grown for two weeks on agar plates in mono-association with the indicated *Lj*-SPHERE (**a**) and *At*-SPHERE (**b**) strains (exp. E). *n* = 6 (3 biologically independent samples × 2 technical replicates). Statistical differences were assessed using a two-sided, non-parametric Mann-Whitney test. Asterisks represent significant values after multiple testing correction using the Benjamini–Hochberg method (*P* < 0.05).

**Extended Data Fig. 7:**
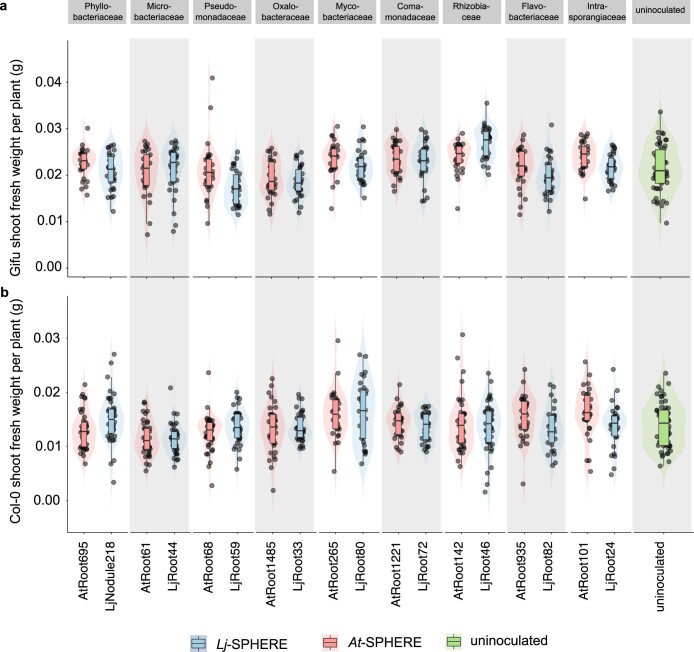
Plant performance in mono-associations. Shoot fresh weight of Gifu (**a**) and Col-0 (**b**) plants grown for two weeks on agar plates in mono-association with the indicated *Lj*-SPHERE and *At*-SPHERE strains (exp. E). *n* = 30 (3 biologically independent samples × 10 technical replicates).

Next, we tested whether communities of commensal bacteria would preferentially colonize roots of their cognate host species (that is, from which they were originally isolated) compared to those of the other host. We found that the aggregated relative abundance of strains from the *Lj*-SPHERE collection was higher in wild-type *Lj* root samples than in those of *At* (Fig. 3e and Extended Data Figs. 4d–f). Likewise, strains from the *At*-SPHERE collection were more abundant on their cognate host than on *Lj*. Commensal host preference and host species community separation was reduced but still present in the *Ljnfr5* mutant (Fig. 3e), suggesting that nodule symbiosis only partially contributes to commensal host preference. Further, sequential in silico removal of individual bacterial families did not alter the observed patterns of host preference at the community level (Extended Data Fig. 5), indicating that host preference was not driven by a single taxonomic group. Mono-association experiments with *Lj* and *At* wild-type plants grown on agar plates revealed that most community members maintained their root colonization capacity, but did not show a significant host preference in isolation (exp. E, Extended Data Fig. 6), suggesting that this commensal phenotype requires a community context. Moreover, we found that shoot biomass of both host species was not affected by these strains, confirming their commensal lifestyle in mono-associations (Extended Data Fig. 7).

**Extended Data Fig. 8:**
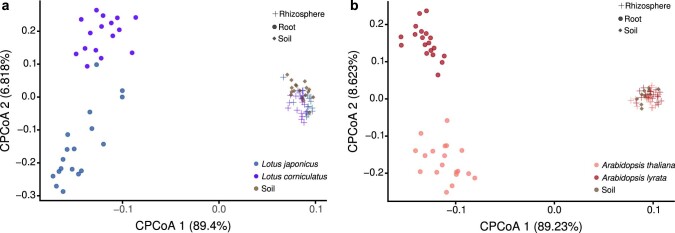
Sister species of *L. japonicus* and *A. thaliana* establish distinct bacterial root communities. Constrained PCoA of Bray-Curtis dissimilarity (constrained by all biological factors and conditioned by all technical variables) of root samples from *L. japonicus* wild type Gifu and *L. corniculatus* (**a**; *n* = 87; variance explained 58.7%, *P* = 0.001), and or root samples from *A. thaliana* wild type Col-0 and *A. lyrata* MN47 (**b**; *n* = 86; variance explained 65%, *P* = 0.001), inoculated and grown with the mixed community *LjAt*-SC3, and of the corresponding rhizosphere and bulk soil communities (exp. F).

We then investigated if the phenotype of commensal host preference was conserved in a plant phylogenetic framework. We selected two additional plant species, *Lotus corniculatus* and *Arabidopsis* *lyrata*, which diverged from *Lj* and *At* approximately 12.5 and 13 Mya, respectively^19,20^, and are indigenous to the region from which the soil used to isolate these bacterial strains was collected^21,22^. We inoculated these four species with a mixed community of *Lj* and *At* commensals and obtained amplicon profiles of root, rhizosphere and unplanted soil samples (exp. F). We observed a significant separation between *Lotus* and *Arabidopsis* root communities (Fig. 3c, *P* = 0.001), and to a lesser extent between samples from the sister species within the same genus (Extended Data Fig. 8), which is in line with similar results obtained from *At* relatives grown in natural sites^23^. We found that the patterns of host preference observed in *Lj* and *At* were retained in their relative species (Fig. 3f), suggesting that this community phenotype might be the result of commensal adaptation to root features conserved in a given host lineage.

### Host factors driving preferred associations in the root microbiota

**Extended Data Fig. 9:**
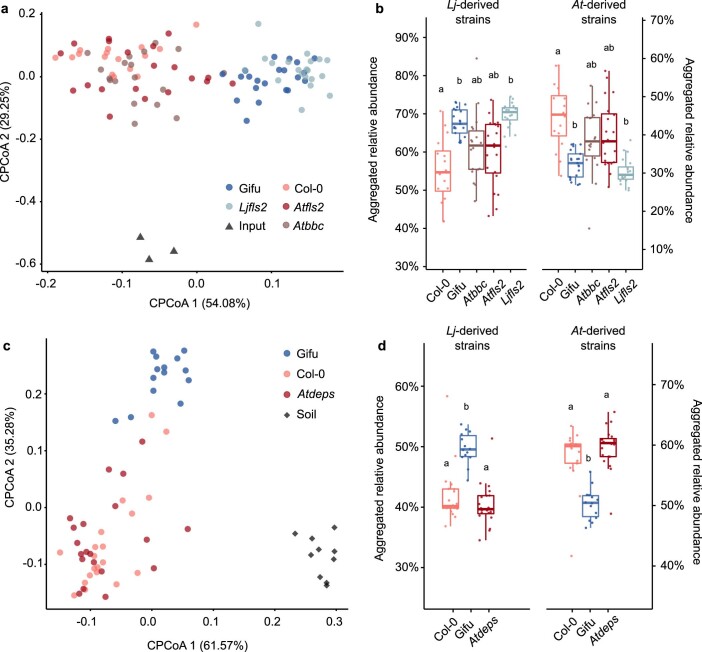
Tested plant immune receptors and signaling pathways do not affect host preference of commensals. **a**, Constrained PCoA of Bray-Curtis dissimilarity (constrained by all biological factors and conditioned by all technical variables; *n* = 98; variance explained 24.6%, *P* = 0.001) of root samples from *L. japonicus* wild type Gifu, *Ljfls2* mutant, *A. thaliana* wild type Col-0, *Atfls2* mutant, and *Atbbc* mutant inoculated and grown with the mixed SynCom *LjAt*-SC1 (exp. G), and of the corresponding bacterial input communities. **b**, Aggregated relative abundance of the 16 *Lj*-derived and the 16 *At*-derived strains in the roots of *Lotus* and *Arabidopsis* plants. *n* = 21 for Gifu, *n* = 16 for Col-0, *n* = 20 for *Ljfls2* and *Atfls2*, *n* = 18 for *Atbbc*. **c**, Constrained PCoA of Bray-Curtis dissimilarity (constrained by all biological factors and conditioned by all technical variables; *n* = 64; variance explained 42.2%, *P* = 0.001) of soil and root samples from Gifu, Col-0, and *Atdeps* mutant inoculated and grown with the mixed SynCom *LjAt*-SC3 (exp. H). **d**, Aggregated relative abundance of the 16 *Lj*-derived and the 16 *At*-derived strains in the roots of *Lotus* and *Arabidopsis* plants. *n* = 14 for Gifu, *n* = 20 for Col-0 and *Atdeps*. *n* refers to biologically independent samples.

Previous studies have reported shifts in *At* leaf or root microbiota structure in mutants impaired in different host immunity pathways^10,24^. We speculated that the plant immune system might also play a role in selecting commensal bacteria in a host-specific manner. We thus tested whether host mutants impaired in perception of ubiquitous microbe-associated molecular patterns (MAMPs) were also preferentially colonized by native commensal strains (exp. G). Community profiles of roots of *At* and *Lj* mutants lacking the receptor FLS2, which detects the bacterial flagellin epitope flg22 (*Ljfls2* and *Atfls2*)^25,26^, were indistinguishable from those of their respective wild types (Extended Data Fig. 9a). Similar results were obtained with an *At* mutant lacking MAMP coreceptors BAK1 and BKK1 as well as CERK1 receptor kinase, known to play a role in the perception of the bacterial MAMP peptidoglycan (*Atbbc* triple mutant)^27^. In addition, bacterial host preference was retained in those mutants (Extended Data Fig. 9b). A separate experiment using the *dde2 ein2 pad4 sid2* (*deps*) mutant in *At*, which is simultaneously defective in all three major defence phytohormone signalling pathways (salicylic acid, jasmonate and ethylene)^28^, showed comparable results (exp. H, Extended Data Fig. 9c,d). Together, these data indicate that the tested MAMP receptors and immune signalling pathways do not play a crucial role in preferential colonization by native commensal bacteria.

**Extended Data Fig. 10:**
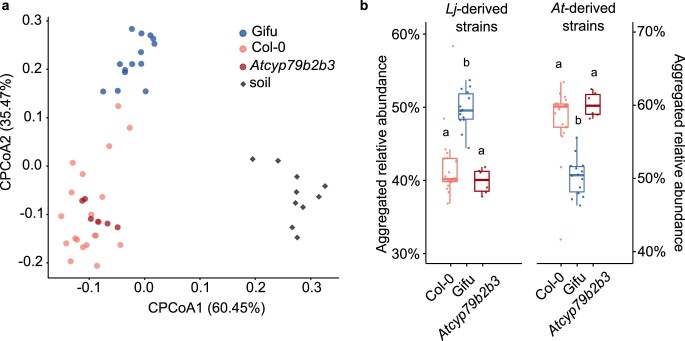
Effect of secreted indole glucosinolates on host preference of commensals. **a**, Constrained PCoA of Bray-Curtis dissimilarity (constrained by all biological factors and conditioned by all technical variables; *n* = 50; variance explained 47.2%, *P* = 0.001) of soil and root samples from *L. japonicus* wild type Gifu, *A. thaliana* wild type Col-0, and *Arabidopsis cyp79b2 cyb79b3* mutant inoculated and grown with the mixed SynCom *LjAt*-SC3 (exp. H). **b**, Aggregated relative abundance of the 16 *Lj*-derived and the 16 *At*-derived strains in the roots of *Lotus* and *Arabidopsis* plants. *n* = 20 for Col-0, *n* = 14 for Gifu, *n* = 6 for *Atcyp79b2b3*. *n* refers to biologically independent samples.

Plant root exudates contain molecular cues that can be differentially metabolized or perceived as signals by root microbiota members^29,30^. In particular, glucosinolates, a group of nitrogen- and sulfur-containing metabolites found in root exudates throughout the family Brassicaceae, including *At*, are known to play a role in plant defence and serve as precursor of compounds that inhibit microbial growth^31–33^. Since legumes such as *Lj* lack genes required for glucosinolate biosynthesis, we speculated that secretion of these compounds by *At* might contribute to the observed differences in community structure. We therefore tested whether the *At cyp79b2 cyp79b3* double mutant^34^, which is defective in the production of microbe-inducible and tryptophan-derived metabolites, including indole glucosinolates, was also preferentially colonized by native commensal strains (exp. H). Comparison of bacterial community profiling data indicates that indole glucosinolate had no effect on overall community structure or bacterial host preference in planta (Extended Data Fig. 10). Notably, incubation of bacterial SynComs in root exudates from *Lj* and *At* plants in an in vitro millifluidics system (exp. I) resulted in small but significant community separation according to the plant genotype (Supplementary Fig. 1a, 5% of variance, *P* = 0.002). However, in this system, we observed a loss of the host preference phenotype (Supplementary Fig. 1b), indicating that root exudates from axenic plants are not sufficient to recapitulate this phenomenon. This observation prompted the question of whether live root tissue was required for preferential colonization by native commensals. We profiled the bacterial communities associated with dead root material from flowering *Lj* and *At* wild-type plants and with inert lignocellulose matrices (softwood birch toothpicks) at 5, 12 and 19 d after inoculation with a mixed community (exp. J). Diversity analyses showed that dead roots and toothpicks harboured distinct microbial communities that were separated from those of soil or detritusphere (soil surrounding dead roots), independently of the timepoint (Fig. 3d). This separation was probably driven by an increase in the relative abundance of Flavobacteria, a taxon associated with the capacity to decompose complex polysaccharides^35^, and which dominates the dead root communities (53% RA on average). Unlike the large separation between living *Lj* and *At* roots (36% of the variance), we observed only a small but significant differentiation between *Lj* and *At* dead root communities (6.4% of variance, *P* = 0.001). Additionally, commensal host preference was undetectable in dead roots, where *Lj*- and *At*-derived strains reached similar aggregated relative abundances in root material harvested from either host (Fig. 3g). Taken together, these results suggest that a living root and other factors besides root exudates, such as a physical contact with the plant (that is, host-commensal feedbacks) are required for host preference in the root microbiota.

### SynCom-specific transcriptional responses of *Lj* and *At* roots

**Fig. 4:**
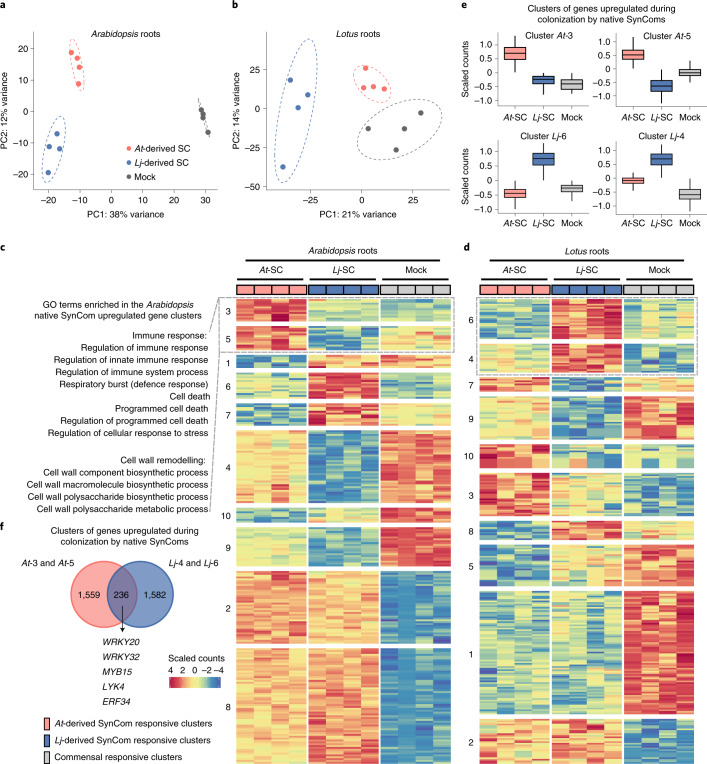
SynCom-specific transcriptional outputs in *Lotus* and *Arabidopsis* roots. **a**,**b**, Whole transcriptome-level principal component analysis of *Arabidopsis* (*n* = 12 biologically independent samples, **a**) and *Lotus* (*n* = 12, **b**) roots after coinoculation with host-specific SynComs (SC) (*Lj*- and *At*-SC3, exp. K). In the case of *Lotus* plants, a nodule isolate from the *Lj*-SPHERE collection was added to all treatments to prevent transcriptional outputs from being dominated by symbiosis or nitrogen starvation responses. **c**,**d**, Heatmaps showing scaled counts of genes arranged according to *k*-means clustering results (only differentially expressed genes shown) for *Arabidopsis* (**c**) and *Lotus* (**d**). **e**, Distribution of expression patterns for clusters of genes upregulated after coinoculation with native SynComs. **f**, Overlap in terms of homologues identified in the same clusters between the two host and a list of relevant transcription factors identified as potential key regulators of differential transcriptional responses. Source data

Next, we sought to assess whether native, non-native or mixed commensal communities elicited a differential response in either host species. We grew wild-type *Lj* and *At* plants in our soil-based gnotobiotic system inoculated with *Lj*-, *At*- or mixed SynComs for 5 weeks (exp. K). Assessment of plant performance revealed that treatment with commensal communities led to increased plant biomass and bacterial load compared to axenic controls, but not to differences according to SynCom treatment (Supplementary Fig. 2). Given the observation that a living root is required for commensal host preference, we conducted RNA-sequencing (RNA-Seq) of cross-inoculated *Lj* and *At* roots to explore host transcriptional responses that might mediate this process (exp. K). Analysis of these data showed that transcriptional outputs separated according to SynCom treatment in both hosts (Fig. 4). Analysis of *k*-means clustering of whole transcriptomes revealed gene clusters associated with general response to bacterial colonization, as well as clusters specific to treatment with native or non-native SynComs. Among genes specifically induced by the native SynComs in both plant hosts we found several transcriptional regulators of immunity (for example, WRKY20, WRKY32 and MYB15), well-characterized MAMP receptor kinases (LYK4) and ethylene response factors (for example, ERF34). This conserved pattern of differential response in the two plant species suggests a specific transcriptional response to native commensal communities that involves components of the host immune system. The differentially expressed transcription factors identified here constitute prime candidates for future exploration of the underlying mechanisms of differential microbiota assembly.

### Invasiveness and persistence in the root microbiota

**Fig. 5:**
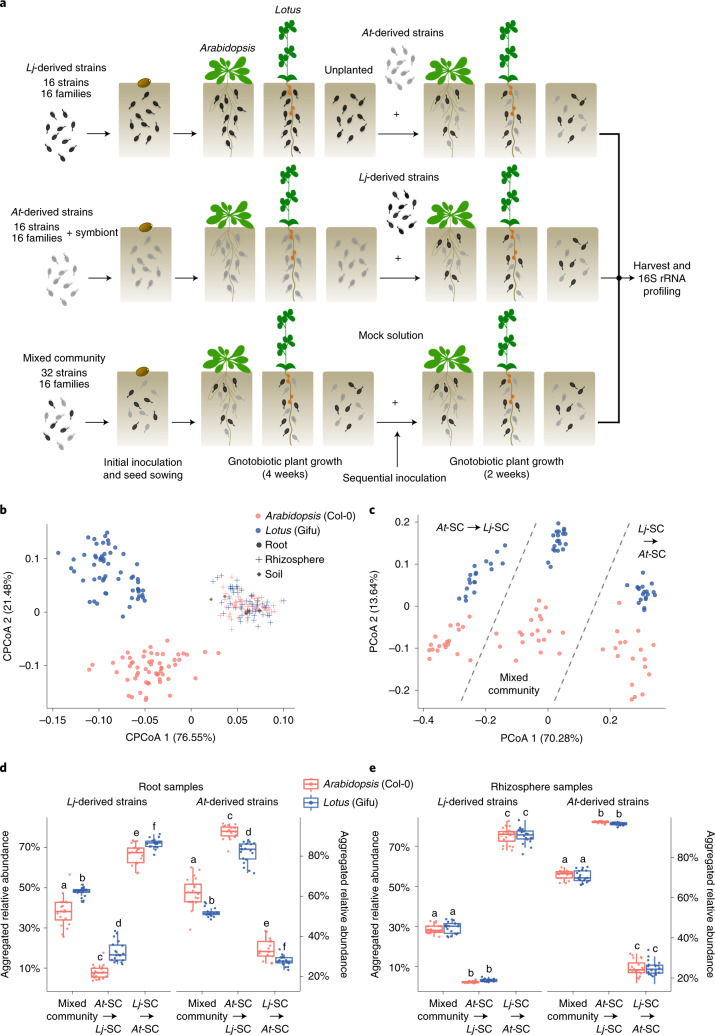
Invasion and persistence of commensal bacteria. **a**, Setup of the sequential inoculation experiment. *Lj* Gifu and *At* Col-0 plants were cocultivated with the mixed community *LjAt*-SC3, or individual SynComs *Lj*-SC3 and *At*-SC3, followed by inoculation with the contrasting SynCom (exp. L). **b**,**c**, Constrained PCoA (CPCoA) of Bray–Curtis dissimilarities (constrained by all biological factors and conditioned by all technical variables; *n* = 267; variance explained 14.7%, *P* = 0.001) of soil, rhizosphere and root samples (**b**), and PCoA of root samples only (*n* = 137, **c**). **d**,**e**, Aggregated RA of the 16 *Lj*-derived and the 16 *At*-derived strains in *Lotus* and *Arabidopsis* root (**d**) (*n* = 120) and rhizosphere (**e**) (*n* = 120) samples in the indicated treatments. Different letters above boxes indicate different significance groups according to a Kruscal–Wallis test, followed by a Dunn’s post hoc. Source data

The results obtained from four independent experiments using five different mixed communities (Fig. 3 and Extended Data Figs. 4, 9 and 10) show that native strains have a competitive advantage when colonizing roots of their cognate host. Ecological theory suggests that in the presence of a competitive hierarchy, the order of species arrival does not matter, as better adapted species tend to dominate irrespective of the history of the community^36^. To investigate the role that priority effects play in root community assembly we designed a series of sequential inoculation experiments using host-specific SynComs (exp. L, Fig. 5a). *At* and *Lj* wild-type plants were inoculated with taxonomically paired SynComs derived from *Lj* (*Lj*-SC3), *At* (*At*-SC3) or a mixed community (*LjAt*-SC3) for 4 weeks. Subsequently, we challenged the established root communities by adding the complementary SynCom (*At*-SC3 or *Lj*-SC3, respectively) to the soil matrix or, in the case of plants initially treated with the mixed community (*LjAt*-SC3), a mock solution (Fig. 5a). We then allowed all plants to grow for an additional 2 weeks before harvesting. Amplicon sequencing showed a significant separation of communities by compartment, and, within root samples, according to host species (Fig. 5b, *P* = 0.001), mirroring the patterns observed in culture-independent community profiles (Fig. 1a). Analysis of beta diversity of root samples at strain-level resolution revealed an effect of the treatment on community structure (Fig. 5c), demonstrating that the order of arrival of strains affects community assembly. Examination of aggregated relative abundances showed that, in a competition context (that is, initial inoculation with the mixed community *LjAt*-SC3), commensal SynComs preferentially colonized roots of their cognate host (Fig. 5d), in line with results from the previous competition experiment shown in Fig. 3. However, in an invasion context, early-arriving SynComs invariably reached higher proportions in the output communities compared to the late-arriving SynComs (Fig. 5d,e). Notably, estimation of absolute bacterial abundances showed that a secondary inoculation with an invading SynCom did not result in a significant increase in total bacterial load (Supplementary Fig. 3). Together, the results from our sequential inoculation experiments (Fig. 5c,d) are indicative of the existence of priority effects in the root and rhizosphere microbiota, a well-known phenomenon in microbial community assembly^36^. These effects could be explained by niche preemption, where early-arriving community members reduce the number of resources available (for example, nutrients, space) for latecomers^37^; alternatively, they could be the result of a feedback process between the host and the early-arriving commensals.

We proposed that commensal bacteria would be less affected by priority effects when colonizing their cognate host, given their competitive advantage with respect to non-native strains. To test this, we examined aggregated relative abundances of *Lj*- and *At*-derived SynComs in the root and rhizosphere communities. We found that host-specific SynComs were better able to invade a resident community in the roots of their cognate host compared to those of the other plant species (Fig. 5d), thus reducing the strength of the priority effects. However, in the rhizosphere compartment of either plant species, host-specific SynComs showed neither host preference in a competition context nor differences in their ability to invade standing communities (Fig. 5e). However, it is also possible that root communities did not reach equilibrium 2 weeks after invasion, and that the observed patterns could change over time.

**Fig. 6:**
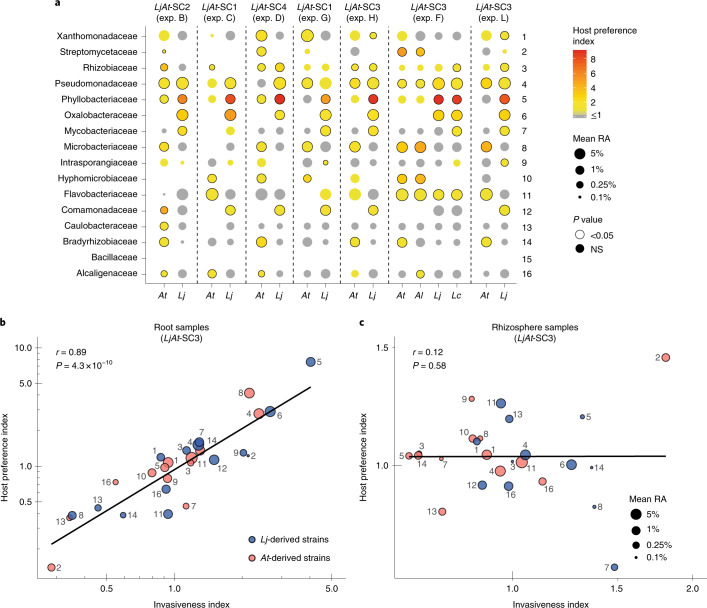
Host preference is linked to invasiveness. **a**, Analysis of host preference of individual commensal strains across gnotobiotic experiments (*n* = 366). Each strain is represented by a dot, whose colour corresponds to its host preference index and whose size to its average relative abundance. A significant host preference (Mann–Whitney test, false-discovery-rate corrected) is depicted by a black circle around a dot. NS, not significant. **b**,**c**, Correlation between host preference and invasiveness index for each strain in root (*n* = 115) (**b**) and rhizosphere samples (*n* = 119) (**c**), respectively, obtained from the sequential inoculation experiment (exp. L). The colour of each point designates the host of origin of each strain and the size denotes its mean relative abundance (log_2_ transformed). Each point is labelled with a numeric identifier that corresponds to the strains in **a** (*LjAt*-SC3). *At*, *A. thaliana*; *Lj*, *L. japonicus*; *Al*, *A. lyrata*; *Lc*, *L. corniculatus*. Source data

We then tested if host preference was directly linked to invasiveness and to what extent these traits were found in individual community members. First, we quantified the strength of host preference by calculating the ratio between the relative abundance of each strain in their cognate host compared to the other plant species (host preference index, Methods). Notably, although *Lj* root samples did not include nodules, but possibly contained incipient symbiotic events, the strains with the highest host preference index were the nitrogen-fixing *Lj* strains belonging to the Phyllobacteriaceae family (Fig. 6a), indicating that host preference of mutualistic rhizobia is not limited to nodule tissue. In addition, multiple other commensal strains showed significant host preference, with members of the families Pseudomonadaceae, Oxalobacteriaceae, Rhizobiaceae and Microbacteriaceae robustly displaying a high host preference index. Members of these last two bacterial families also had an impact on community structure during invasion in a recent study with phyllosphere bacteria^12^. Next, we calculated an invasiveness index by comparing the ability of each strain to invade a standing community on their cognate host compared to the other plant species (Methods). We found a strong correlation between host preference and invasiveness of commensal bacteria, which is independent of their relative abundance (*r* = 0.89, *P* = 4.3 × 10^−10^; Fig. 6b). In contrast, this correlation was absent in the rhizosphere samples (Fig. 6c), indicating that the link between these two bacterial traits is mediated by host attributes that do not extend to the rhizosphere. Together, our data show that host preference is prevalent in commensal bacteria from diverse taxonomic groups and that this trait is tightly linked to invasiveness and together play a role during root microbiota assembly.

## Discussion

The current concept of host specificity in plant–microbe interactions was originally developed based on studies using microorganisms with either pathogenic or mutualistic lifestyles. Recently, it has been shown that soilborne, nitrogen-fixing *Ensifer meliloti* mutualists can adapt to local host genotypes in only five plant generations and proliferate to greater abundances in hosts with shared evolutionary histories^38^. We show here that in the *Lj* and *At* root microbiota, there is a gradient of host preference among commensals belonging to diverse taxonomic lineages. Maintenance of host preference in the sympatric relative species *L. corniculatus* and *A. lyrata* raises the possibility that these commensals might have adapted to host features conserved in the respective plant genera. Alternatively, the observed host preference patterns might be the consequence of other ecological processes, such as ecological fitting, whereby organisms are able to colonize and persist in a new environment using traits that they already possess^39^. Diversification of plant traits as a result of adaptation to edaphic or other environmental factors is expected to result in new host features that constitute new root niches for microbial colonization. It is also possible that host diversification is partly driven by the adaptation of plants to commensal microbiota in soils with contrasting properties. However, the observation that in our experimental conditions colonization by native or non-native bacterial SynComs had no impact on plant growth suggests that host preference is the result of microbial adaptation to host features instead of coevolution. However, it is possible that a significant impact on host fitness might be observed in long-term experimentation, or in the presence of biotic or abiotic stresses, which were absent in the tested conditions, or in direct competition with other plant species. This latter hypothesis is supported by the observation that similarity between the root microbiota of different species affects competitive plant–plant interactions and has an impact on host performance through plant–soil feedback^5^. Future experimentation using multi-species gnotobiotic systems and varying environmental conditions will serve to test these hypotheses.

In aquatic and terrestrial ecosystems, microbial traits such as growth rate, antagonistic activity or resource use efficiency are known determinants of invasiveness^40,41^. In microbial communities associated with eukaryotic organisms, the ability to interact with the host might also be required for successful invasion. Our results indicate that native commensals have a competitive advantage when invading standing communities in the root but not in soil or rhizosphere. One possibility is that increased invasiveness by native bacteria is enabled by the existence of unfilled host-species-specific root niches that can be occupied by latecomers. Alternatively, direct interaction of commensals with their host may be required to trigger the formation of host-species-specific root niches, which could be linked to the specific transcriptional reprogramming in roots observed during colonization by native SynComs. This latter hypothesis is further supported by the observation that bacterial SynComs colonizing dead roots or incubated in root exudates in vitro showed no significant host preference. Our study provides a framework to test these hypotheses and to investigate the molecular basis of host preference in multiple taxa of the bacterial root microbiota in comparison with host adaptation mechanisms in plant pathogens and mutualists.

## Methods

### Bacterial and plant material

Bacterial strains were grown in tryptic soy broth (15 g l^−1^, TSB, Sigma-Aldrich) liquid medium or on agar plates containing 15 g l^−1^ of Bacto Agar (Difco) at 25 °C. *Mesorhizobium* strains LjNodule210, LjNodule215 and LjNodule218, isolated from *Lj* root nodules, were cultured in TY medium (5 g l^−1^ tryptone, 3 g l^−1^ yeast extract) supplemented with 10 mM CaCl_2_ or in YMB medium (5 g l^−1^ mannitol, 0.5 g l^−1^ yeast extract, 0.5 g l^−1^ K_2_HPO_4_·3H_2_O, 0.2 g l^−1^ MgSO_4_·7H_2_O, 0.1 gl l^−1^ NaCl). The composition of synthetic bacterial communities (SynComs) is listed in Supplementary Table 1. *Lj* ecotype Gifu B-129 was used as wild-type. Symbiosis-deficient mutant *nfr5-2* (ref. ^18^) and flagellin receptor-deficient mutant *fls2* (LORE1-30003492)^25^ were derived from the Gifu B-129 genotype. For *At*, ecotype Columbia-0 was used as wild-type. Mutant genotypes *fls2* (ref. ^26^), *bbc*^27^, *deps*^28^ and *cyp79b2 cyb79b3* (ref. ^34^) were available in our seed stock. *L. corniculatus* seeds, cultivated in the North-Western German lowland, were retrieved from Rieger-Hofmann GmbH, Blaufelden-Raboldshausen, Germany. *A. lyrata* MN47 seeds were a gift from J. de Meaux, University of Cologne.

### Establishment of the *Lj* bacterial culture collection

The *Lj* culture collection combines strains isolated during three independent isolation events. Bacterial isolation, DNA isolation and identification using Illumina sequencing were performed as previously described^8^. Wild-type *Lj* (ecotype Gifu B-129) plants were grown in natural soil (Cologne agriculture soil (CAS), batch 10 from spring 2014 and batch 11 from spring 2015) in the greenhouse and harvested after 4 or 8 weeks to cover different developmental stages. Root systems of 20 plants were subjected to DNA isolation and culture-independent community profiling via amplicon sequencing. From 45 plants, a 4-cm section of the roots was collected and rigorously washed three times with phosphate-buffered saline (130 mM NaCl (7.6 g l^−1^), 7 mM Na_2_HPO_4_ (1.246 g l^−1^), 3 mM NaH_2_PO_4_ (0.414 g l^−1^), pH 7.0) and three times with sterile water. Nodule and root parts were separated and homogenized independently. Homogenized roots from each individual plant were allowed to sediment for 15 min and the supernatant was diluted (1:20,000, 1:40,000 and 1:60,000) with four different media: 3 g l^−1^ TSB, 50% TY, Casitone yeast for enrichment of Myxococcales (3 g l^−1^ Casitone, 1.36 g l^−1^ CaCl_2_·2H_2_O and 1 g l^−1^ yeast extract; pH adjusted to 7.2) and yeast agar van Niel’s, for enriching of Burkholderiales (10 g l^−1^ yeast extract, 1 g l^−1^ K_2_HPO_4_ and 0.5 g l^−1^ MgSO_4_·7H_2_O). Bacterial dilutions cultivated in 96-well microtitre plates. Homogenized nodules from each individual plant were directly diluted (1:20,000, 1:40,000 and 1:60,000) and cultivated in 96-well microtitre plates. This procedure was carried out for individual plants to obtain bacterial isolates from different plant roots. After 10–20 d at room temperature, plates that showed visible bacterial growth in around 30 wells were chosen for high-throughput sequencing. Bacterial isolates were identified with a two-step barcoded PCR protocol described previously^8^, with the difference that at the first step of the PCR, the v5-v7 fragments of the 16S rRNA gene were amplified by the degenerate primers 799F (AACMGGATTAGATACCCKG) and 1192R (ACGTCATCCCCACCTTCC), and indexing was done using Illumina-barcoded primers. The indexed 16S rRNA amplicons were pooled, purified and sequenced on the Illumina MiSeq platform. Strains isolated from nodules were tested for their ability to form functional nodules in *Lj* Gifu plants grown on agar plates.

Cross-referencing of IRL sequences with culture-independent profiles was used to identify candidate strains for further characterization, purification and WGS. Two main selection criteria were used: maximum taxonomic coverage, selecting candidates from as many taxa as possible and priority to strains whose 16S sequences were highly abundant in the natural communities. Whenever multiple candidates from the same phylogroup were identified, we aimed to obtain multiple independent strains, if possible, coming from separate biological replicates to ensure they represented independent isolation events. After validation of selected strains, 294 (including nine isolated from nodules) were successfully subjected to WGS.

For WGS, DNA was isolated from strains using the QiAmp Micro DNA kit (Qiagen), treated with RNase and purified. Quality control, library preparation and sequencing (on the Illumina HiSeq3000 platform) were performed by the Max Planck Genome Centre, Cologne, Germany (https://mpgc.mpipz.mpg.de/home/). Sequencing depth was 5 million reads per sample.

### Culture-independent community profiling

Bacterial communities were profiled by amplicon sequencing of the variable v5-v7 regions of the bacterial 16S rRNA gene. Library preparation for Illumina MiSeq sequencing was performed as described previously^2^. In all experiments, multiplexing of samples was performed by double-indexing (barcoded forward and reverse oligonucleotides for 16S rRNA gene amplification).

### Greenhouse experiment

*Lj* Gifu and *At* Col-0 were grown for 5 weeks in CAS soil (batch 15 from January 2020) in 7 × 7 cm pots alongside unplanted control pots under short-day conditions. Pots were watered with sterile water from the bottom as needed. Root, rhizosphere and soil samples were harvested and processed as described previously^42^. In total, 15, 13 and eight replicates were sampled for Col-0, Gifu and unplanted controls, respectively. DNA was isolated from those samples using the MP Biomedicals FastDNA Spin Kit for Soil.

### Multi-species microbiota reconstitution experiments

We used the gnotobiotic FlowPot system^2,17^ to grow *At* and *Lj* plants with and without bacterial SynComs. In brief, the system allows for even inoculation of each growth pot with microbes by the flushing of pots with the help of a syringe attached to the bottom opening. Sterilized seeds are placed on the matrix (peat and vermiculite, 2:1 ratio), and pots are incubated under short-day conditions (10 h light, 21 °C; 14 h dark, 19 °C), standing in customized metal racks in sterile plastic boxes with filter lids (SacO2 microboxes, www.saco2.com). For SynCom preparation, bacterial commensals were grown separately in liquid culture for 2–5 d to reach high density, harvested and washed in 10 mM MgSO_4_. Equivalent amounts of each strain were combined to yield the desired SynComs with an optical density (OD_600_) of 1. An aliquot of 200 µl of the SynCom as reference sample for the experiment start, and aliquots of 50 µl of the individual strains were taken and stored at −80 °C for sequencing. The SynCom was added to the desired medium to reach a final OD_600_ of 0.02. FlowPots were each flushed with 50 ml of inoculum (medium/SynCom mix). Generally, the medium used for inoculation was 0.25× B&D^43^ supplemented with 1 mM KNO_3_ for both plant species. In experiments D, F, K and M (Supplementary Table 2), 0.5× MS (2.22 g l^−1^ Murashige+Skoog basal salts, Duchefa; 0.5 g l^−1^ MES anhydrous, BioChemica; adjusted to pH 5.7 with KOH) was used for *Arabidopsis*. The two plant species were grown in separate FlowPots side-by-side, with ten pots in total per plastic box. After 5 weeks of growth, roots were harvested and cleaned thoroughly from attached soil using sterile water and forceps. *Lotus* root segments containing nodules were omitted. Soil samples from planted and unplanted pots were collected as rhizosphere and soil samples, respectively. All root (epiphytic and endophytic compartments), rhizosphere and soil samples were transferred to Lysing Matrix E tubes (FastDNA Spin Kit for Soil, MP Biomedicals), frozen in liquid nitrogen and stored at −80 °C for further processing. DNA was isolated from those samples using the FastDNA Spin Kit for Soil, and from individual strains of the SynCom via quick alkaline lysis^8^, then subjected to bacterial community profiling or absolute quantification of bacteria. For RNA isolation, samples were harvested the same way and processed using the RNeasy Plant Mini kit (Qiagen).

### Dead root experiment

Mature root systems from Gifu and Col-0 plants grown in potting soil in the greenhouse were harvested from flowering plants (13-week old *Lotus*, 7-week old *Arabidopsis*), washed several times in water, padded on kitchen paper to remove moisture and dried in big glass petri dishes at 120 °C for 1 h. Note that Gifu plants had a few small, most probably ineffective root nodules. Pieces of the dried, dead roots were planted into FlowPots under sterile conditions, and SynCom (*LjAt*-SC3) inoculation was performed as described above. Dead roots were recovered from the FlowPots after 5, 12 and 19 d of incubation, and washed and stored as described above for live roots.

### SynCom invasion experiments

FlowPots were sequentially inoculated with native and non-native strains. FlowPots were prepared as usual, with the addition of a round nylon filter (pore size 200 µm) at the bottom of the pot to avoid clogging of the bottom opening by matrix material. FlowPots were first inoculated with the mixed SynCom (16 *Lj*- and 16 *At*-strains), the *At* SynCom (16 *At*-strains), the *Lj* SynCom (16 *Lj* strains) or the mock solution (medium only). The medium used for inoculation was 0.25× B&D^43^ supplemented with 1 mM KNO_3_ for both plant species.

For sterilization, *At* seeds were incubated for 5 min in 70% ethanol, then twice for 1 min in 100% ethanol, washed five times with sterile water and stored at 4 °C in the dark for stratification. *Lj* seeds were scarified by abrading the surface using sand paper, incubated for 20 min in diluted bleach and washed five times with sterile water. Sterilized seeds were placed on sterile Whatman paper wetted with sterile water in a squared petri dish and allowed to germinate under short-day conditions. Sterilized Col-0 seeds and germinated sterile Gifu seeds were placed on the soil surface. Note that a few drops of *Mesorhizobium* culture (*Lotus* root nodule symbiont, strain LjNodule218, OD_600_ 0.02) were applied to Gifu seedlings in the *At* SynCom treatment to allow for normal root nodule symbiosis to occur and ensure healthy plant growth. After growth for 4 weeks, a second inoculation was performed, where a mock inoculum (medium) was added to the mixed SynCom-treated pots, the *Lj* SynCom was added to the *At* SynCom-treated pots, the *At* SynCom was added to the *Lj* SynCom-treated pots and mock inoculum was added to the mock-treated pots. The pots were flushed in reverse by adding the inoculum from the top and applying vacuum from the bottom. On a sterile bench, FlowPots (cut 60-ml syringes with a male Luer Lok connector) were placed onto female Luer Lok connectors of a vacuum manifold (QIAvac 24 Plus, Qiagen), keeping the valves of the manifold closed. Vacuum was applied to the manifold with an attached vacuum pump. Next, 20 ml of inoculum were carefully added to a pot with a 20-ml syringe and needle, avoiding damage of the plant shoots. Each pot was inoculated by opening and closing the corresponding valve. Pots were put back into the plastic containers and plants grown for another 2 weeks. Root, rhizosphere and soil samples were harvested as described above.

### Collection of root exudates

*Arabidopsis* and *Lotus* plants were grown in a customized hydroponic system (original design by M. Peukert, University of Cologne, unpublished). This sterile growth setup consists of glass jars filled with glass beads and a stainless-steel mesh on top. Nutrient solution (modified 0.25× B&D medium, Fe-EDTA instead of Fe-citrate) was poured into the jars until the beads were covered in liquid and the liquid touched the metal mesh. We used the same medium for both plant species to allow for direct comparison of exudate composition, and to minimize differential effects on the bacterial community originating from different media types. We chose the *Lotus* B&D medium since *Arabidopsis* grew reasonably well in it. Sterilized and pregerminated seeds were placed onto the mesh, jars were put into sterile plastic boxes with filter lids (SacO2 microboxes) and plants were grown for 5 weeks. The medium containing root exudates was removed from the jars in the clean bench using a sterile metal needle and plastic syringe. After transfer to 50-ml Falcon tubes, exudates were frozen at −80 °C, freeze-dried until a volume of 2–3 ml was left, thawed and adjusted with sterile water to 5 ml. Exudates were kept at −80 °C until further usage.

### Millifluidics experiment

Bacterial incubation in root exudates was performed in a millifluidics system (MilliDrop Analyzer, MilliDrop, www.millidrop.com). This drop-based system allows incubation of bacteria in very small volumes of root exudates or growth medium. In brief, bacteria and exudates or growth medium are combined in wells of a 96-well plate using a pipetting robot Freedom Evo 100 (Tecan). Droplets of approximately 100–200 nl are then sucked in from the wells of the loading plate by a tip on the robotic arm of the MilliDrop Analyzer, generating hundreds of droplets within an oil-filled tube, separated by air spacers. During incubation, the droplet ‘train’ moves back and forth, so that during each round, each droplet passes a detector that counts the droplets. Culture droplets are collected after the experiment and subjected to community profiling.

The mixed community *LjAt*-SC1 was used and was essentially prepared as described above for the in planta experiments, adjusted to OD_600_ of 0.1 and used as input for preparation of the loading plate. Pure exudates (pH between 7.0 and 8.0) or a defined M9 + carbon growth medium (1× M9 salts including phosphate buffer, 1 mM magnesium sulfate, 0.3 mM calcium chloride, 1× vitamin B solution and artificial root exudates, pH 7.0) was used for incubation. Vitamin B solution contained 0.4 mg l^−1^ 4-aminobenzoic acid, 1 mg l^−1^ nicotinic acid, 0.5 mg l^−1^ calcium-d-pantothenate, 1.5 mg l^−1^ pyridoxine hydrochloride, 1 mg l^−1^ thiamine hydrochloride, 0.1 mg l^−1^ biotin and 0.1 mg l^−1^ folic acid (modified from ref. ^44^). Artificial root exudates (modified from ref. ^45^) were composed of 0.9 mM glucose, 0.9 mM fructose, 0.2 mM sucrose, 0.8 mM succinic acid, 0.6 mM sodium lactate, 0.3 mM citric acid, 0.9 mM serine, 0.9 mM alanine and 0.5 mM glutamic acid. Bacteria were incubated for 3 d, during which the pH of the cultures stayed stable. Droplets were collected in 6-µl amounts, and DNA isolated via quick alkaline lysis^8^, which consisted of addition of 10 µl of buffer 1 (25 mM NaOH, 0.2 mM EDTA, pH 12), incubation at 95 °C for 30 min, addition of 10 µl of buffer 2 (40 mM Tris-HCl at pH 7.5) and storage at −20 °C.

### Mono-associations of SynCom members with host plants

*Lotus* seeds were sterilized and placed on sterile wet Whatman paper for germination. Seedlings were transferred to squared petri dishes containing 0.25× B&D medium (with Fe-EDTA instead of Fe-citrate) supplemented with 3 mM KNO_3_ and 1% Difco Bacteriological agar, and sterile filter paper was put on top of the sloped solidified medium before placing the seedlings to prevent root growth inside the agar. *Arabidopsis* seeds were sterilized and germinated on 0.5× MS medium plus 1% Difco Bacteriological agar. Seedlings were transferred to squared petri dishes containing 0.5× MS medium (neutral pH, buffered with 2 mM HEPES) plus 1% agar. The 32 strains of the mixed community *LjAt*-SC3 were grown individually in liquid medium, harvested and adjusted to an OD_600_ of 0.02. Seedlings were inoculated by adding 500 µl of bacterial culture to the roots. Plants were grown for 14 d under long-day conditions (16/8 day–night cycles) at 21 °C. Three biological replicates were prepared for each genotype–bacteria combination.

### Absolute quantification of bacteria

Genomic DNA was isolated from roots of plants grown in FlowPots (experiments K and L). DNA concentration was determined with the Quant-iT PicoGreen double-stranded DNA Assay Kit (Thermo Fisher Scientific). To quantify bacterial load on plant roots, the amount of bacterial DNA relative to the amount of plant DNA was determined via quantitative PCR (qPCR). For bacteria, the v5-v7 region of the 16S rRNA gene was amplified using the AACMGGATTAGATACCCKG (799F) and ACGTCATCCCCACCTTCC (1192R) primers. For Col-0, a fragment of At1g12360 was amplified using the TCCGGTCAATATTTTTGTTCG and TATAGCAGCGAAAGCCTCGT primers, and for Gifu, a fragment of the *NFR5* gene was amplified using the TCATATGATGGAGGAGTTGTCTGTT and ATATGAGCTTCGGAGCATGG primers. qPCR was performed as described previously^46^. The amount of 16S rRNA was normalized to plant gene within each individual sample using the following equation: 16S rRNA gene over plant gene = 2^-*C*t(16S)^/2^-*C*t(plant)^.

For colony counts (exp. E), roots were harvested, washed, weighed and crushed in 500 µl (Col-0) or 750 µl (Gifu) of sterile water. Serial dilutions of 10^−1^, 10^−2^, 10^−3^, 10^−4^ and 10^−5^ of the crushed roots were prepared in sterile water. Then 10 µl of each were spotted onto 10% TSB agar square plates. Single colonies were counted after 1–3 d.

### Processing of 16S rRNA gene amplicon data

Amplicon sequencing data from *Lj*^15^ and *At*^2^ roots of plants grown in CAS soil in the greenhouse, along with unplanted controls, were demultiplexed according to their barcode sequence using the QIIME^47^ pipeline. DADA2 (ref, ^48^) was used to process the raw sequencing reads of each sample. Unique amplicon variants (ASVs) were inferred from error-corrected reads, followed by chimera filtering, also using the DADA2 pipeline. ASVs were aligned to the SILVA database^49^ for the taxonomic assignment using the naïve Bayesian classifier implemented by DADA2. Raw reads were mapped to the inferred ASVs to generate a relative abundance table, which was subsequently used for analyses of diversity and differential abundance using the R package vegan^50^.

Amplicon sequencing reads from the *Lotus* and *Arabidopsis*^8^ IRLs and from their corresponding culture-independent root community profiling were quality-filtered and demultiplexed according to their two-barcode (well and plate) identifiers using custom scripts and a combination of tools included in the QIIME^47^ and USEARCH^51^ pipelines. Sequences were clustered into OTUs with a 97% sequence identity similarity using the UPARSE algorithm, followed by identification of chimeras using UCHIME^52^. Samples (wells) with fewer than 100 good quality reads were removed from the data set as well as OTUs not found in a well with at least ten reads. A purity threshold of 90% was chosen for identification of recoverable OTUs. We identified *Lj*-IRL samples matching OTUs found in the culture-independent root samples and selected a set of 294 representative strains maximizing taxonomic coverage for subsequent validation and WGS, forming the basis of the core *Lj*-SPHERE collection.

Sequencing data from SynCom experiments (including FlowPot and millifluidics experiments) were preprocessed similarly as natural community 16S rRNA data. Quality-filtered, merged paired-end reads were then aligned to a reference set of sequences extracted from the whole-genome assemblies of every strain included in a given gnotobiotic experiment, using USEARCH (uparse_ref command)^53^. Only sequences with a perfect match to the reference database were retained. We checked that the fraction of unmapped reads did not significantly differ between compartment, experiment or host species. We generated a count table that was used for downstream analyses of diversity with the R package vegan^50^. We visualized amplicon data from all experimental systems using the ggplot2 R package^54^.

### Host preference and invasiveness indices

To quantify the strength of the host preference of each bacterial strain individually, we calculated the ratio between the mean relative abundance of a given SynCom member in root samples of their cognate host and its mean relative abundance in root samples of the other plant species. The host preference indices depicted in Fig. 5a were calculated independently for each experiment. To avoid obtaining very high ratios due to small denominator values, strains with mean relative abundances below 0.1% in either of the two hosts were removed from the analysis. Similarly, an invasiveness index was calculated by obtaining the ratio between mean relative abundance of a strain when invading resident communities on roots of their cognate host, compared to the other plant species. The invasiveness index was calculated using samples from the sequential inoculation experiment (experiment L, Fig. 4). The direct comparison between the two indices shown in Fig. 5b,c were calculated using samples from experiment L only, where invasion and competition treatments were performed in parallel. To test whether a SynCom member was significantly more abundant in the roots of their cognate host (that is, significant host preference), we used the non-parametric Wilcoxon test controlling for false-discovery rate with *α* = 0.05.

### Bacterial genome assembly and annotation

Paired-end Illumina reads were first subjected to length-trimming and quality-filtering using Trimmomatic^55^. Reads were assembled using the A5 assembly pipeline^56^, which uses the IDBA algorithm^57^ to assemble error-corrected reads. Detailed assembly statistics and corresponding metadata can be found in Supplementary Data 2. Genomes with multi-modal *k*-mer and GC content distributions or multiple instances of marker genes from diverse taxonomic groups were flagged as not originating from clonal cultures. These samples were processed using a metagenome binning approach^58^. Briefly, contigs from each metagenome sample were clustered using METABAT2 (ref. ^59^), followed by an assessment of completeness and contamination of each metagenome-assembled genome using CheckM^60^. Only bins with completeness scores larger than 75% and contamination rates lower than 5% were retained and added to the collection (Supplementary Data 2, designated metagenome-assembled genome (MAG) in the column ‘type’). Functional annotation of genes was conducted using Prokka and using a custom database based on Kyoto Encyclopedia of Genes and Genomes (KEGG) orthologue groups^61^ downloaded from the KEGG FTP server in November 2019. Hits to sequences in the database were filtered using an *E* value threshold of 10 × 10^−9^ and a minimum coverage of 80% of the length of the query sequence.

### Phylogenomic analysis of the *Lj*- and *At*-SPHERE culture collections

Genomes from the *Lj*- and *At*-SPHERE culture collections^8^ were searched for the presence of a set of 31 conserved, single-copy marker genes, known as AMPHORA^62^ genes. Sequences of each gene were aligned using Clustal Omega^63^ with default parameters. Using a concatenated alignment of each gene, we inferred a maximum likelihood phylogeny using FastTree^64^. We visualized this tree using the Interactive Tree of Life web tool^65^. Genomes from both collections (*Lj*-SPHERE and *At*-SPHERE) were clustered into phylogroups, roughly corresponding to a species designation^66^ using FastANI^67^ and a threshold of average nucleotide identity at the whole-genome level of at least 97%.

### RNA-Seq and data analysis

RNA isolated from FlowPot samples was subjected to quality control, library preparation and sequencing (on the Illumina HiSeq3000 platform) at the Max Planck Genome centre, Cologne, Germany (https://mpgc.mpipz.mpg.de/home/). Sequencing depth was 6 million reads per sample.

Raw Illumina RNA-Seq reads were preprocessed using fastp (v.0.19.10)^68^ with default settings for pair-end reads. High-quality reads were pseudo-aligned to the *Lj* Gifu or *At* Col-0 transcriptome reference using kallisto (v.0.46.1)^69^. After removal of low abundant transcripts that were not present in at least two replicates under each condition, count data were imported using the tximport package^70^.

Differential expression analyses were performed using the DESeq2 package^71^. First, raw counts were normalized with respect to the library size (rlog function) and transformed into log_2_ scale. We tested for sample effects by surrogate variable analysis using the sva package^72^. Significant surrogate variables were automatically detected and integrated into the model for differential analyses. Principal component analysis based on whole transcripts were then conducted and plotted to visualize the cluster and variance of biological replicates under each condition. Transcripts with fold-changes >1.5 and adjusted *P* value for multiple comparisons (Benjamini–Hochberg method) equal to or below 0.05 were considered significant.

The log_2_ scaled counts were normalized by the identified surrogate variables using the limma package^73^ (‘removeBatchEffect’ function), and transformed as median-centred *z*-score (by transcripts, ‘scale’ function). Then *z*-scores was used to conduct *k*-means clustering for all transcripts. The cluster number (*k* = 10) was determined by sum of squared error and Akaike information criterion. Differential expressed transcripts and cluster results were visualized using heatmaps generated by ComplexHeatmap package^74^.

Gene ontology enrichment for each cluster using the whole *Lotus* and *Arabidopsis* transcriptomes as backgrounds were performed with the goseq package^75^, which considers the transcripts length bias in RNA-Seq data. Gene ontology annotations were retrieved from the Gene Ontology Consortium (September 2019)^76,77^. Significantly changed biological process Gene ontology terms (adjusted *P* < 0.05) were visualized in dot plots using the clusterProfiler package^78^.

### Statistics and reproducibility

All experiments were performed with full factorial (biological and technical) replication. Competition experiments using SynComs were in addition repeated multiple times (Extended Data Fig. 1) using independent bacterial communities. Whenever bacterial abundances or plant growth parameters were compared, we used a two-sided, non-parametric Mann–Whitney test or, in the case of multiple comparisons, a Kruskal–Wallis test, followed by a Dunn’s post hoc. Whenever appropriate, *P* values were adjusted for multiple testing using the Benjamini–Hochberg method (*α* = 0.005). Statistical tests on beta-diversity analyses were performed using a PERMANOVA test with 5,000 random permutations. Whenever boxplots were used in figures, data were represented as median values (horizontal line), Q1 − 1.5× interquartile range (boxes) and Q3 + 1.5× interquartile range (whiskers).

### Reporting Summary

Further information on research design is available in the Nature Research Reporting Summary linked to this article.

## Supplementary information

Supplementary Information Supplementary Note, Figs. 1–3 and Tables 1 and 2.

Reporting Summary

Supplementary Data 1 Data and metadata of *Lj*- and *At*-IRLs.

Supplementary Data 2 Metadata of the *Lj*- and *At*-SPHERE core culture collection.

Supplementary Data 3 Data and metadata of *LjAt* SynCom experiments.

## Data Availability

The strains of the *Lj*-SPHERE collection will be deposited at and will be available on request from the Leibniz Institute DSMZ in Braunschweig, Germany. Raw 16S rRNA amplicon reads have been deposited in the European Nucleotide Archive under the accession number PRJEB37695. Similarly, sequencing reads and genome assemblies of the *Lj*-SPHERE core collection have been uploaded to the same database with the accession number PRJEB37696. Source data are provided with this paper.

## Source data

Source Data Fig. 1 Statistical source data.
Source Data Fig. 2 Statistical source data.
Source Data Fig. 3 Statistical source data.
Source Data Fig. 4 Statistical source data.
Source Data Fig. 5 Statistical source data.
Source Data Fig. 6 Statistical source data.

## Code availability

The scripts used for the computational analyses described in this study are available at http://www.github.com/garridoo/ljsphere, to ensure replicability and reproducibility of these results.
