# Supplementary material for: Host preference and invasiveness of commensal bacteria in the Lotus and Arabidopsis root microbiota
Source: Nat Microbiol. 2021 Jul 26;6(9):1150–62. doi: 10.1038/s41564-021-00941-9 (PMC8387241; doi:10.1038/s41564-021-00941-9)
Supplement: Supplementary file 1 — Supplementary Note, Figs. 1–3 and Tables 1 and 2. [file 41564_2021_941_MOESM1_ESM.pdf]

nature portfolio

https://doi.org/10.1038/s41564-021-00941-9

Supplementary information

Host preference and invasiveness of commensal bacteria in the *Lotus* and *Arabidopsis* root microbiota

In the format provided by the authors and unedited

Host preference and invasiveness of commensal bacteria in the *Lotus* and *Arabidopsis* root microbiota

Kathrin Wippel[1]†, Ke Tao[2]†, Yulong Niu[1], Rafal Zgadzaj[1], Niklas Kiel[3], Rui Guan[1], Eik Dahms[1], Pengfan Zhang[1], Dorthe B. Jensen[2], Elke Logemann[1], Simona Radutoiu[2]*, Paul Schulze-Lefert[1,3]*, Ruben Garrido-Oter[1,3]*

Correspondence to: garridoo@mpipz.mpg.de, schlef@mpipz.mpg.de, radutoiu@mbg.au.dk

This PDF file includes the following Supplementary Information:

Supplementary Note
Supplementary Figures 1 to 3
Supplementary Tables 1 and 2

Supplementary Note

Culture collection recovery rates

To explore the mechanisms by which different plant species assemble distinct microbial communities, we established a taxonomically and functionally diverse culture collection of the *Lotus* root and nodule microbiota (Methods). A total of 3,960 colony-forming units (CFUs) were obtained and taxonomically characterized by sequencing the bacterial *16S* ribosomal RNA (rRNA; Supplementary Data 1), resulting in a comprehensive sequence-indexed rhizobacterial library from *L. japonicus* (*Lj*-IRL). In parallel, a subset of the root samples was also subjected to amplicon sequencing to obtain culture-independent community profiles for cross-referencing with the *Lj*-IRL data. Recovery rates were estimated by calculating the number of bacterial OTUs (Operational Taxonomic Units, defined by 97% sequence identity) found in the natural communities that had at least one isolate in our culture collection (Methods). For *Lotus*, the recovery rates varied between 50% (based on the top 100 most abundant OTUs), 53% (OTUs with RA ≥ 0.1%), and 64.58% (prevalent OTUs, found in at least 80% of the natural community samples). Recovered OTUs accounted for up to 82% of the cumulative relative abundance of the entire culture-independent community (Fig. 1c), indicating that our collection is representative of a large fraction of the *Lotus* root microbiota. By comparison, the recovery rates for the *A. thaliana* culture collection (*At*-IRL) varied between 51% (top 100 OTUs), 57% (≥ 0.1% relative abundance), and 62.82% (prevalent OTUs), while recovered OTUs recovered from *Arabidopsis* roots reached a cumulative relative abundance of 59% of the entire community (Fig. 1e). Interestingly, 45.57% of the abundant OTUs found in the natural communities of *Lj* roots were recovered in the *At*-IRL, whereas 45.19% of abundant OTUs from *At* roots were recovered in the *Lj*-IRL (Fig. 1d, and 1f). These results are indicative of a substantial overlap of the recovered bacterial OTUs.

Taxonomic and functional overlap of the *Lotus* and *Arabidopsis* culture collections

To establish a core *Lotus* culture collection of whole-genome sequenced strains (*Lj*-SPHERE), we selected from the *Lj*-IRL a taxonomically representative subset of bacterial isolates maximizing the number of taxa covered (Methods). A total of 294 isolates belonging to 20 families and 124 species, including both commensal and symbiotic bacteria, were subjected to whole-genome sequencing (Supplementary Data 2). This core collection is of a similar size and diversity as the collection from *Arabidopsis* roots (*At*-SPHERE)[8]. A whole-genome phylogeny of all sequenced isolates from both collections revealed an extensive taxonomic overlap between exemplars derived from *Lotus* and *Arabidopsis* (Fig. 2), indicating that the observed differences in natural community structures (Fig. 1b) are likely not driven by the presence of host-specific bacterial taxonomic groups. Instead, the distinct root community profiles of the two hosts are possibly due to differences in the relative abundance of shared taxonomic groups (Extended Data Fig. 2).

We hypothesized that bacterial preference for a plant species should be accompanied by the acquisition of a set of genes required for preferential colonization of a specific host. In order to test this, we characterized the functional potential encoded in the genomes of the sequenced isolates using the KEGG orthology database as a reference[61]. We observed that a large proportion of annotated gene families was shared between the two culture collections (6,712 out of 7,456), and that the number of gene families exclusively found in genomes of strains derived from *Lotus* or *Arabidopsis* roots (3.51% and 6.47%, respectively) did not significantly deviate from what would be expected by chance ($P = 0.49$). However, additional host-specific genes are likely encoded in sequences for which a functional annotation is currently unavailable (~27%). Principal coordinates analysis (PCoA) of functional distances revealed a high degree of overlap between isolates of the same taxonomic groups, which was independent

of their host of origin (Extended Data Fig. 3). Permutation analysis of variance confirmed that the main driver of functional variation encoded in the genomes of our culture collections was the taxonomy of the isolates (79.70% of variance explained; *P* = 0.001), and that the origin of isolation (i.e., host species) only explained a small fraction of the functional diversity encoded by these genomes (4.27% of variance; *P* = 0.001).

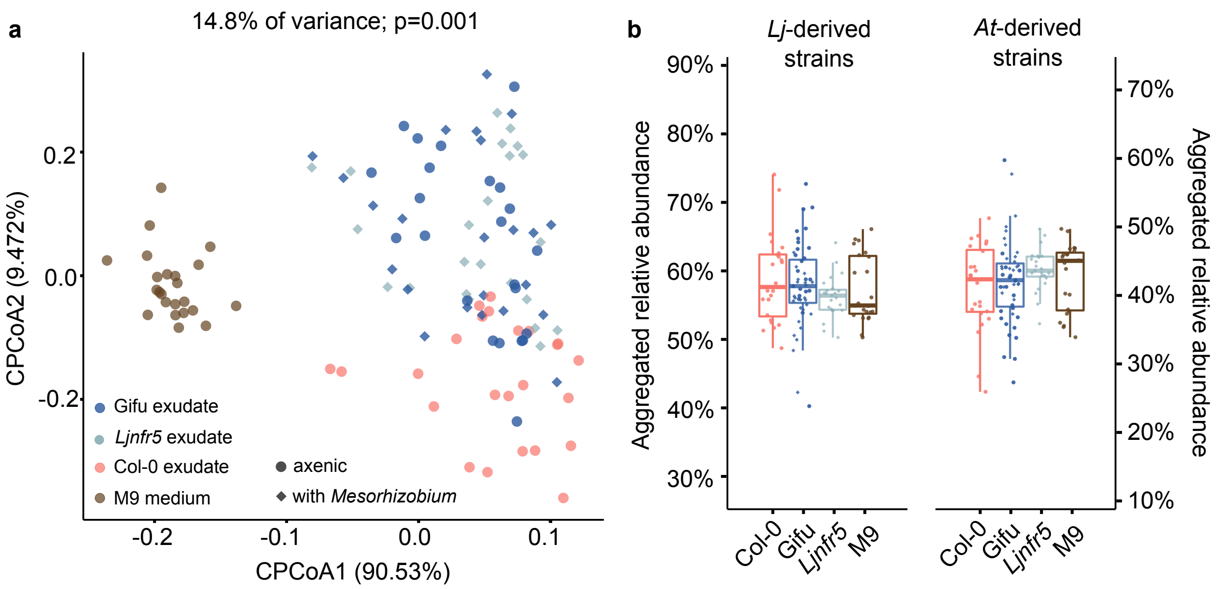

Supplementary Figure 1 │ Effect of soluble root exudates on host preference of commensals. a, Constrained PCoA of Bray-Curtis dissimilarity (constrained by all biological factors and conditioned by all technical variables; *n* = 116) of the mixed SynCom *LjAt*-SC1 incubated in root exudates from axenically grown Gifu or Col-0, from Gifu or *Ljnfr5* inoculated with the symbiont *Mesorhizobium*, or in a carbon-rich control medium M9 (exp. I). **b**, Aggregated relative abundance of the 16 *Lj*-derived and the 16 *At*-derived strains in the *Lotus* and *Arabidopsis* exudates. A Kruskal-Wallis test showed no significant differences in the distribution of values among groups. *n* = 24 for Col-0, *n* = 47 for Gifu, *n* = 23 for *Ljnfr5* and M9. *n* refers to biologically independent samples.

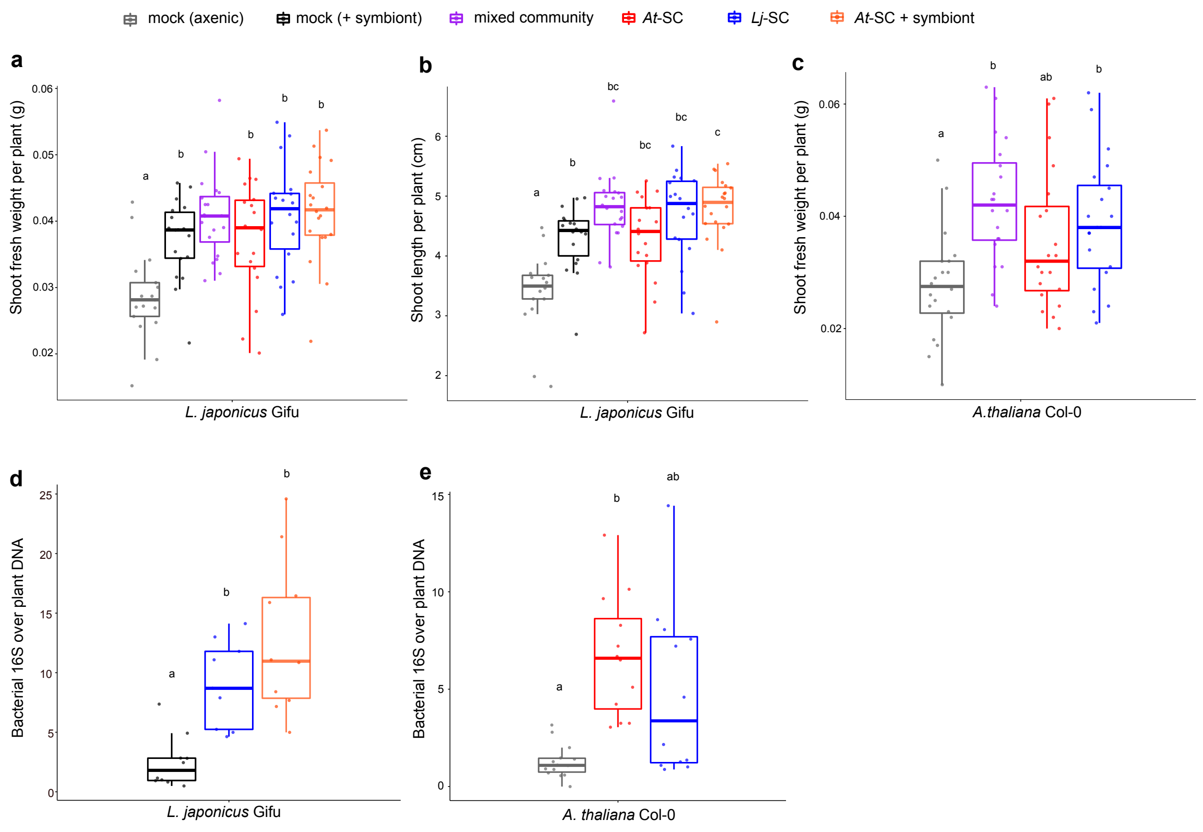

Supplementary Figure 2 | Shoot phenotypes of *Lotus* and *Arabidopsis* plants inoculated with different commensal communities. *L. japonicus* Gifu and *A. thaliana* Col-0 plants were co-cultivated with the mixed community *LjAt*-SC3, or individual SynComs *Lj*-SC3 and *At*-SC3 (exp. K). Shoot fresh weight of *Lotus* (**a**) and *Arabidopsis* (**c**), as well as shoot length of *Lotus* (**b**) were measured after five weeks. Bacterial load on Gifu (**d**) and Col-0 (**e**) roots was quantified *via* qPCR. Each data point corresponds to one replicate comprising roots of 2-4 plants grown in the same pot. Shared letters indicate no significant difference based on Kruskal-Wallis and Wilcoxon rank sum test ($P < 0.05$). In **a** and **b**, $n = 17$ for mock (axenic), $n = 18$ for mock (+symbiont), for *At*-SC and *Lj*-SC, $n = 20$ for mixed community and *At*-SC +symbiont. In **c**, $n = 20$ for all conditions. In **d**, $n = 10$ for all conditions. In **d**, $n = 14$ for mock, $n = 12$ for *Lj*-SC and *At*-SC. *n* refers to biologically independent samples. A Kruskal-Wallis test followed by a Dunn's *post hoc* was used to assess significant differences in the distribution of values among groups ($P < 0.05$).

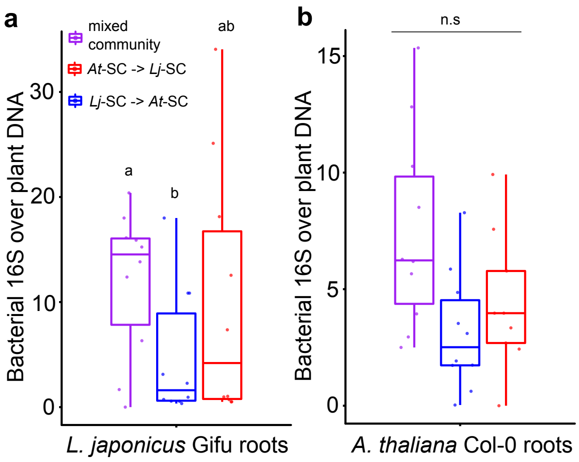

Supplementary Figure 3 | Quantification of bacterial load on plant roots after sequential inoculation with native and non-native commensals. *L. japonicus* Gifu and *A. thaliana* Col-0 plants were co-cultivated with the mixed community *LjAt*-SC3, or individual SynComs *Lj*-SC3 and *At*-SC3, followed by inoculation with the remaining SynCom (exp. L). Colors relate to the early-arriving community. Amount of 16S rRNA gene copies relative to plant gene copies as proxy for bacterial load on *Lotus* (**a**) and *Arabidopsis* (**b**) roots is shown. Each data point corresponds to one replicate comprising roots of 2-4 plants grown in the same pot. A Kruskal-Wallis test followed by a Dunn's *post hoc* was used to assess significant differences in the distribution of values among groups ($P < 0.05$, $n = 10$ biologically independent samples for each condition in **a** and **b**).

Supplementary Table 1 | Bacterial SynComs used in this study

Class	Family	*LjAt*-SC1		*LjAt*-SC2		*LjAt*-SC3		*LjAt*-SC4		*LjAt*-SC5	
		At-SC1	*Lj*-SC1	*At*-SC2	*Lj*-SC2	*At*-SC3	*Lj*-SC3	*At*-SC4	*Lj*-SC4	*At*-SC5	*Lj*-SC5
Betaproteobacteria	Alcaligenaceae	AtRoot83	LjRoot1	AtRoot170	LjRoot1	AtRoot83	LjRoot1	AtRoot83	LjRoot1	AtRoot83	LjRoot1
Firmicutes	Bacillaceae	AtRoot131	LjRoot5	AtRoot11	LjRoot53	AtRoot131	LjRoot5	AtRoot131	LjRoot5	AtRoot147	LjRoot15
Alphaproteobacteria	Bradyrhizobiaceae	AtRoot123D2	LjRoot52	AtRoot123D2	LjRoot4	AtRoot123D2	LjRoot52	AtRoot123D2	LjRoot52	AtRoot670	LjRoot90
Alphaproteobacteria	Caulobacteraceae	AtRoot77	LjRoot17	AtRoot1290	LjRoot17	AtRoot77	LjRoot17	AtRoot77	LjRoot17	AtRoot655	LjRoot284
Betaproteobacteria	Comamonadaceae	AtRoot404	LjRoot109	AtRoot16D2	LjRoot72	AtRoot1221	LjRoot72	AtRoot1221	LjRoot72	AtRoot29	LjRoot20
Bacteroidetes	Flavobacteriaceae	AtRoot186	LjRoot149	AtRoot935	LjRoot82	AtRoot935	LjRoot82	AtRoot935	LjRoot82	AtRoot901	LjRoot82
Alphaproteobacteria	Hyphomicrobiaceae	AtRoot436	LjRoot16	AtRoot436	LjRoot3	AtRoot685	LjRoot16	AtRoot685	LjRoot16	AtRoot635	LjRoot222
Actinobacteria	Intrasporangiaceae	AtRoot85	LjRoot27	AtRoot101	LjRoot24	AtRoot101	LjRoot24	AtRoot101	LjRoot24	AtRoot563	LjRoot49
Actinobacteria	Microbacteriaceae	AtRoot61	LjRoot44	AtRoot4	LjRoot42	AtRoot61	LjRoot44	AtRoot61	LjRoot44	AtRoot53	LjRoot12
Actinobacteria	Mycobacteriaceae	AtRoot265	LjRoot80	AtRoot135	LjRoot80	AtRoot265	LjRoot80	AtRoot265	LjRoot80	AtRoot135	LjRoot80
Betaproteobacteria	Oxalobacteraceae	AtRoot335	LjRoot35	AtRoot1485	LjRoot33	AtRoot1485	LjRoot33	AtRoot1485	LjRoot33	AtRoot418	LjRoot25
Alphaproteobacteria	Phyllobacteriaceae	AtRoot695	LjNodule218	AtRoot554	LjNodule210	AtRoot695	LjNodule218	AtRoot695	LjNodule218	AtRoot157	LjNodule215
Gammaproteobacteria	Pseudomonadaceae	AtRoot71	LjRoot54	AtRoot68	LjRoot59	AtRoot68	LjRoot59	AtRoot68	LjRoot59	AtRoot569	LjRoot154
Alphaproteobacteria	Rhizobiaceae	AtRoot142	LjRoot46	AtRoot142	LjRoot2	AtRoot142	LjRoot46	AtRoot142	LjRoot46	AtRoot73	LjRoot11
Actinobacteria	Streptomycetaceae	AtRoot63	LjRoot303	AtRoot1295	LjRoot303	AtRoot1310	LjRoot303	AtRoot1310	LjRoot303	AtRoot431	LjRoot303
Gammaproteobacteria	Xanthomonadaceae	AtRoot480	LjRoot21	AtRoot627	LjRoot60	AtRoot480	LjRoot60	AtRoot480	LjRoot60	AtRoot559	LjRoot143
Sphingomonadales	Sphingomonadaceae									AtRoot720	LjRoot262
Actinobacteria	Cellulomonadaceae							AtRoot137			
Gammaproteobacteria	Moraxellaceae							AtRoot1280			
Actinobacteria	Nocardiaceae							AtRoot136			
Actinobacteria	Nocardioidaceae							AtRoot224			
Actinobacteria	Promicromonosporaceae							AtRoot22			
Betaproteobacteria	Burkholderiaceae								LjRoot22		
Actinobacteria	Micrococcaceae								LjRoot78		

present in *LjAt*-SC1
present in *LjAt*-SC2
present in all mixed SynComs
present in *LjAt*-SC3 and *LjAt*-SC4
members of host-specific families
present in *LjAt*-SC5

LjAt-SC1 was used in experiments C, G, and I (see Supplementary Table 2).

LjAt-SC2 was used in experiment B, (full-factorial replicate of C) to comprise independent strains of the same families, as far as possible (distinguishable 16S sequence).

LjAt-SC3 was built using strains from *LjAt*-SC1 and *LjAt*-SC2 to generate an idependent community.

LjAt-SC4 is identical to *LjAt*SC3, but includes strains from host-specific bacterial families.

LjAt-SC5 was used in experiment M, (full-factorial replicate of D) to comprise independent strains of the same families.

In general, mixed communites were designed to include strains distinguishable based on *16S* rRNA gene sequence, to have a similar number of strains in the *Lj* and *At* SynCom, to consist of taxonomically paired *Lj* and *At* SynComs (so that any differences in community structure would be attributable to the origin of strain isolation, i.e., the host plant). In addition, the SynCom design was influenced by practical constraints, e.g., not all strains of the current culture collection or their genome sequences were available at the

Supplementary Table 1 | Bacterial SynComs used in this study

Supplementary Table 2 | List of experiments

ID	Sequencing run ID	Growth or incubation system	Treatments	2nd inoculation (at 4 weeks)	Genotypes	Compartments harvested	No. of samples for sequencing	Growth period	Analysis
A	AtLj_009	greenhouse pots with CAS soil	CAS soil		Gifu	roots	13	5 weeks	16S profiling
						rhizosphere	13		
					Col-0	roots	15		
						rhizosphere	15		
					unplanted	soil	8		
B	AtLj_002	gnotobiotic FlowPots with sterilized potting soil	*LjAt*-SC2		Gifu	root	24	5 weeks	16S profiling
						rhizosphere	24		
					Col-0	root	19		
						rhizosphere	24		
					Ljnfr5	root	23		
						rhizosphere	24		
					unplanted	soil	18		
C	AtLj_001	gnotobiotic FlowPots with sterilized potting soil	*LjAt*-SC1		Gifu	root	24	5 weeks	16S profiling
						rhizosphere	24		
					Col-0	root	21		
						rhizosphere	22		
					Ljnfr5	root	23		
						rhizosphere	23		
					unplanted	soil	18		
D	AtLj_006	gnotobiotic FlowPots with sterilized potting soil	*LjAt*-SC4		Gifu	root	18	5 weeks	16S profiling
						rhizosphere	18		
					Col-0	root	16		
						rhizosphere	15		
					unplanted	soil	20		
E	n.a.	agar plates	individual strains of *LjAt*-SC3		Gifu	roots	n.a	2 weeks	CFU counts for bacterial load, shoot fresh weight
					Col-0	roots	n.a	2 weeks	
F	AtLj_007	gnotobiotic FlowPots with sterilized potting soil	*LjAt*-SC3		Gifu	root	18	5 weeks	16S profiling
						rhizosphere	18		
					Col-0	root	19		
						rhizosphere	19		
					L. corniculatus wild type	root	16		
						rhizosphere	17		
					A. lyrata MN47	root	19		
						rhizosphere	19		
					unplanted	soil	28		
G	AtLj_003	gnotobiotic FlowPots with sterilized potting soil	*LjAt*-SC1		Gifu	root	21	5 weeks	16S profiling
					Col-0	root	16		
					Ljfls2	root	20		
					Atfls2	root	20		
					Atbbc	root	18		
H	AtLj_005	gnotobiotic FlowPots with sterilized potting soil	*LjAt*-SC3		Gifu	root	14	5 weeks	16S profiling
					Col-0	root	20		
					Atdeps	root	20		
					Atcyp79b2 Atcyp79b3	root	6		
					unplanted	soil	10		
I	MDA10	MilliDrop millifluidics	*LjAt*-SC1		Gifu (exudates)	droplets	47	3 days	16S profiling
					Col-0 (exudates)	droplets	24		
					Ljnfr5 (exudates)	droplets	23		
					M9 medium	droplets	23		

Supplementary Table 2 | List of SynCom experiments

Supplementary Table 2 cont. | List of experiments

ID	Sequencing run ID	Growth or incubation system	Treatments	2nd inoculation (at 4 weeks)	Genotypes	Compartments harvested	No. of samples for sequencing	Growth period	Analysis
J	AtLj_008	gnotobiotic FlowPots with sterilized potting soil	*LjAt*-SC3		Gifu (dead roots)	dead root	45	5, 12, 19	16S profiling
						detritusphere	45		
					Col-0 (dead roots)	dead root	44		
						detritusphere	45		
					toothpick	wood	35		
					unplanted	soil	36		
K	n.a.	gnotobiotic FlowPots with sterilized potting soil	*LjAt*-SC3		Gifu	roots	n.a.	5 weeks	qPCR for bacterial load, shoot fresh weight, RNA seq
					Col-0	roots	n.a.		
			Lj-SC3		Gifu	roots	n.a.		
					Col-0	roots	n.a.		
			At-SC3		Gifu	roots	n.a.		
					Col-0	roots	n.a.		
			mock		Gifu	roots	n.a.		
					Col-0	roots	n.a.		
L	AtLj_004	gnotobiotic FlowPots with sterilized potting soil	*LjAt*-SC3	mock	Gifu	root	20	6 weeks	16S profiling, qPCR for bacterial load
						rhizosphere	20		
					Col-0	root	20		
						rhizosphere	20		
					unplanted	soil	10		
			Lj-SC3	*At*-SC3	Gifu	root	10		
						rhizosphere	20		
					Col-0	root	20		
						rhizosphere	20		
					unplanted	soil	10		
			At-SC3	*Lj*-SC3	Gifu	root	19		
						rhizosphere	19		
					Col-0	root	19		
						rhizosphere	20		
					unplanted	soil	10		
M	AtLj_010	gnotobiotic FlowPots with sterilized potting soil	*LjAt*-SC5		Gifu	root	20	5 weeks	16S profiling
						rhizosphere	20		
					Col-0	root	20		
						rhizosphere	20		
					unplanted	soil	20		

Gifu, *L. japonicus* wild type; Col-0, *A. thaliana* wild type; SC, synthetic community (see also Supplementary Table 1).

Supplementary Table 2 cont. | List of SynCom experiments
